# Porcine Single-Eye Retinal Pigment Epithelium Cell Culture for Barrier and Polarity Studies

**DOI:** 10.3390/cells14131007

**Published:** 2025-07-01

**Authors:** Philipp Dörschmann, Sina von der Weppen, Emi Koyama, Johann Roider, Alexa Klettner

**Affiliations:** Department of Ophthalmology, University Medical Center, Kiel University, Arnold-Heller-Str. 3, Haus B2, 24105 Kiel, Germanyalexa.klettner@uksh.de (A.K.)

**Keywords:** 3R principle, best practice protocol, primary cell culture, retinal pigment epithelium, polarity, barrier, tight junctions, age-related macular degeneration, single-eye culture, Transwell

## Abstract

Age-related macular degeneration (AMD) is the main cause of blindness in Western nations. AMD models addressing specific pathological pathways are desired. Through this study, a best-practice protocol for polarized porcine single-eye retinal pigment epithelium (RPE) preparation for AMD-relevant models of RPE barrier and polarity is established. Single-eye porcine primary RPE cells (from one eye for one well) were prepared in 12-well plates including Transwell inserts. Different coatings (laminin (Lam), Poly-ᴅ-Lysine (PDL), fibronectin (Fn) and collagens) and varying serum contents (1%, 5% and 10%) were investigated to determine optimal culture parameters for this model. Success rates of cultures, cell number (trypan-blue exclusion assay), morphology/morphometry (light and fluorescence microscopy), protein secretion/expression (ELISA, Western blot), gene expression (qPCR), transepithelial electric resistance (TEER) and polar location of bestrophin 1 (BEST1) by cryosectioning (IHC-Fr) were assessed. Cells seeded on Lam exhibited the highest level of epithelial cells and confluence properties. Fn resulted in the highest cell number growth. Lam and Fn exhibited the highest culture success rates. TEER values and vascular endothelial growth factor secretion were highest when Lam was used. For the first time, polar (Transwell) porcine single-eye RPE morphometry parameters were determined. RPE on Lam showed bigger cells with a higher variety of cell shapes. CIV displayed the lowest claudin 19 expression. The highest basolateral expression of BEST1 was achieved with Lam coating. The higher the serum, the better the cell number increase and confluence success. A reduction in serum on Lam showed positive results for RPE morphology, while morphometry remained stable. A five percent serum on Lam showed the highest culture success rate and best barrier properties. RPE65 expression was reduced by using 10% serum. Altogether, the most suitable coating of Transwell inserts was Lam, and a reduction in serum to 5% is recommended, as well as a cultivation time of 28 days. A protocol for the use of polar porcine single-eye cultures with validated parameters was established and is provided herein.

## 1. Introduction

**Figure 1:**
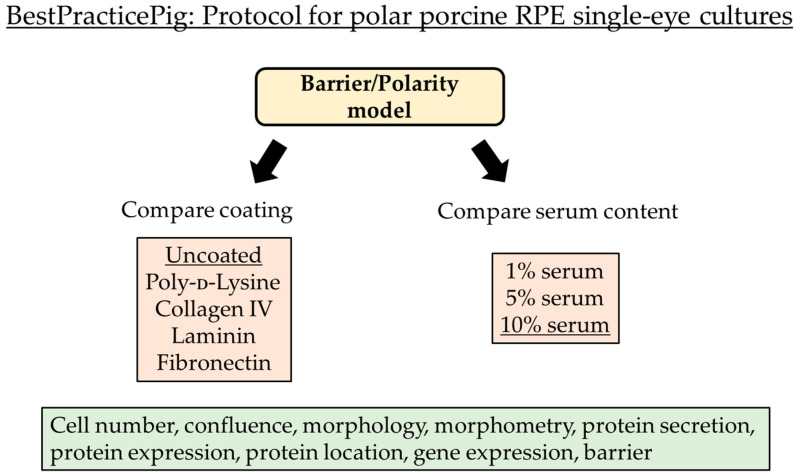
Flowchart of experimental schedule. The aim of this study is to provide a model system for polar porcine retinal pigment epithelium single-eye cultures (yellow). Cells of individual pig eyes are seeded into different wells of a 12-well plate with a Transwell insert generating genetic homogenous polar cultures per well. Coating and serum contents of the media were compared (light red). The conducted biological tests are listed (green). Typical standard preparation parameters are underlined and are used as reference controls for this study. RPE = retinal pigment epithelium.

The demand for excellent cell culture models for deciphering retinal diseases featuring the retinal pigment epithelium (RPE) is high. The RPE is located between the photoreceptors and the choroid, fulfilling many functions to maintain the photoreceptors and to uphold visual function [1]. The RPE supplies nutrients for and discards the waste of the photoreceptors, phagocytoses shed photoreceptor outer segments, recycles the visual pigments, releases protective cytokines and protects against light and oxidative stress [2]. An important function of the RPE is to form the outer blood–brain barrier, protecting the photoreceptors from blood-derived cells and factors, and contributing to the immune privilege of the retina [3]. The RPE is involved in many retinal diseases, most importantly in the development of age-related macular degeneration (AMD) [1], the most relevant disease causing central vision loss in Western nations [4]. In order to investigate RPE cells, several models are regularly used in research. The cell line ARPE-19, patient-derived pluripotent stem cells, human fetal RPE cells, or primary cultures from mice are examples of commonly used models [5]. Each model system has its advantages and limits. ARPE-19 cells are fast and easy to handle [6]; however, under widely used culture conditions, ARPE-19 cells hardly show an RPE phenotype and differ strongly in their gene expression [7,8]. In addition, they generally do not develop a functioning barrier [9]. Patient-derived pluripotent stem cells have the benefit to be of human origin; however, hPSC-derived RPEs display subpopulations with different types of gene expression and maturity within a single culture and can undergo epigenetic modification, which is absent in natural RPEs [10,11,12]. The use of human fetal RPE cells has strong ethical implications and may be legally strictly regulated. In addition, fetal RPEs differ from adult RPEs in various aspects, such as their gene expression or degree of barrier formation [5]. And the RPE of mice displays some intriguing differences compared to human RPE, such as the expression of different claudins in their tight junctions, a higher degree of multi-nucleated cells and a generally low barrier [3,13]. A model that closely resembles the adult human RPE is primary RPE cells derived from adult porcine eyes. Porcine eyes resemble the human eye as closely as a non-primate possibly can [5]. The pig is a diurnal animal with eyes of high similarity in anatomy and size compared to humans. It mainly uses cone vision and its retina contains the area centralis, an area of higher visual acuity. These cones are similar to the humans’ blue, red and green cones, and it shows a similar level of multi-nucleation and types of claudins in tight junctions compared to humans [3,14,15]. A general drawback of porcine RPE cell culture is a higher variability compared to cell lines or mice strains [5]. Also, RPE cell culture methods vary between labs. Furthermore, standard RPE cell cultures are mixed cultures, combining RPE cells of different donor eyes in one dish [5]. In order to reduce the limitations of porcine RPE cell culture, we have recently published a best-practice protocol for porcine single-eye RPE cell culture in 12-well plates (non-polar) [16]. In this model, RPEs from one pig eye are seeded into 1 well of a 12-well plate, resulting in genetically equal cells in one culture, and genetic diversity between the cultures, mimicking the real-world situation more closely. Furthermore, this model ensures a more unified response of the cells to a given stimulus within one culture. The anticipated higher inter-donor variability can be matched by a higher sample size. In the published protocol, we establish standards for cultivation, such as post-mortem time, coating, and serum content, and we are the first to describe standard morphometric parameters of porcine RPEs [16]. However, our published culture was optimized for standard non-polar 12-well cultures without barrier formation. In this study, we have amended our best-practice protocol for porcine single-eye RPE culture on Transwell plates to study barrier and polarity (Figure 1), investigating the effect of serum and different coatings. Cell culture coating can have a profound effect on the characteristics of cultured RPE cells, concerning adhesion, cell division, differentiation, and function [17,18,19]. Importantly, it has been shown that the coatings can influence the barrier of RPE cells [20,21]. Our results suggest that laminin and 5% serum should be used, and we present standard morphometric data for polarized porcine RPE cells.

## 2. Materials and Methods

### 2.1. Polar Single-Eye Retinal Pigment Epithelium Preparation

Primary porcine RPEs were prepared from pigs’ eyes (Deutsche Landrasse, Duroc, hybrid; mixed sex; half a year old) 4 to 5 h post-mortem. They were obtained from animals whose eyes are removed before being processed in food production; these animals are also checked by veterinarians. The use of porcine eyes in research was approved by the Animal Welfare Officer of University Kiel according to the German Animal Welfare Act (TierSchG, https://www.gesetze-im-internet.de/tierschg (accessed on 6 February 2025)). It is not considered as animal experiment, but contributes to a reduction in animal use in science (3R Principle).

**Figure 2:**
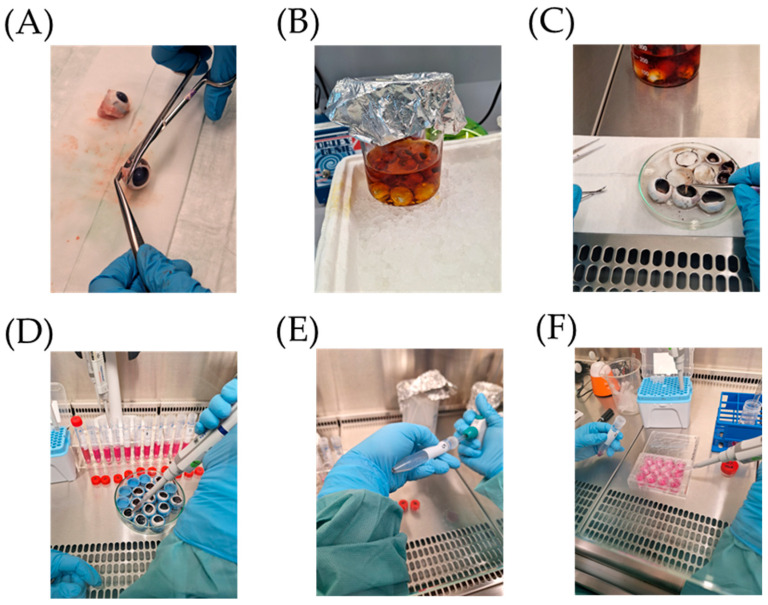
Polar single-eye RPE preparation from porcine eyes. (**A**) Cleaning eyes from adjacent tissue, (**B**) storing eyes in cold NaCl, (**C**) opening eyes and removing vitreous body and retina, (**D**) trypsinization of eyes, (**E**) washing and centrifugation, and (**F**) seeding cells on Transwell inserts.

The preparation in a single-eye format (cells from one eye into one well) was established and a detailed protocol was described that can be used [16]. In brief, non-necessary tissue was cut off and the organs were treated with cold betaisodona (Mundipharma, Germany; Frankfurt am Main, Germany; #04923204), followed by washing in cold 0.9% NaCl (Fresenius Kabi, Bad Homburg, Germany; #04801702). Eyes were cut open and retina were removed in Dulbecco’s phosphate-buffered saline without Ca^2+^ and Mg^2+^ (DPBS, Pan-Biotech, Aidenbach, Germany; #P04-53500) containing 1% penicillin/streptomycin (Pe/St, Sigma-Aldrich, St. Louis, MO, USA; #P0781). Eyes were filled with 37 °C warm 0.25% trypsin (Pan-Biotech; #P10-021100) and 0.25% trypsin/0.02% ethylenediaminetetraacetic acid (EDTA, Pan-Biotech; #P10-020100) for 10 and 35 min, respectively. Cells were washed two times with 5 mL media consisting of Dulbecco’s Modified Eagle Medium with high glucose, L-glutamine, phenol red (DMEM, Gibco™, Thermo Fisher Scientific, Waltham, MA, USA; #41965062), 11 mM sodium pyruvate (Pan-Biotech; #P04-43100), 1% non-essential amino acids (Pan-Biotech; #P08-32100), 1% Pe/St and 10% fetal bovine serum (FBS Mexican origin, Gibco™, Thermo Fisher Scientific; #10437028, Lot: 2405703RP). Cells were resuspended in 1 mL media and seeded into individual wells of 12-well plates (Sarstedt, Nümbrecht, Germany; #83.3921), with 1 well left without cells for blank barrier measurements with 0.4 µm Transwell inserts (Sarstedt; #83.3931.041) and kept at 37 °C and 5% CO_2_. In the Transwell inserts, 1 mL of media was applied apically and 1.5 mL was applied basolaterally. The preparation process is demonstrated in Figure 2. For serum testing, serum content of media was changed from 10% to 5% or 1%, during the first feeding after three days of cultivation. To assess barrier function, transepithelial resistance was measured with the EVOM3 Epithelial Volt/Ohm (TEER) Meter (World Precision Instruments Germany GmbH, Friedberg, Germany) 7, 10, 14 and 28 days after preparation. All other parameters were measured at days 7, 14 and 28 after preparation. The time points were chosen according to our findings for non-polar single-eye RPE cell culture [16] and preliminary findings.

To count cells before seeding and after cultivation, they were detached with trypsin/EDTA. A total volume of 20 µL cell suspension was blended with 20 µL 0.4% trypan blue solution (Sigma-Aldrich; #T8154) followed by 3 min of incubation. The solution was pipetted to a Neubauer chamber (Carl Roth GmbH + Co. KG, Karlsruhe, Germany, #T729.1). Viable cells were determined with the microscope Axiovert 160 (Carl Zeiss AG, Oberkochen, Germany) with 10× magnification. Change in cell number was calculated as the difference in cell number at time points with respect to the seeding cell count from the same well.

### 2.2. Coating of Transwell Inserts

Different coating agents were used to coat individual Transwell membranes (polyester, 1.1 cm^2^). Collagen Type IV (CIV, Advanced Biomatrix, Inc., Carlsbad, CA, USA; #5022), Poly-ᴅ-Lysine (PDL, Sigma-Aldrich; #P7886), laminin (Lam, Sigma-Aldrich; #11243217001) or fibronectin (Fn, Advanced Biomatrix; #5080) were used as described in the corresponding instructions with recommended doses (CIV—100 µg/mL; PDL—100 µg/mL; Lam—20 µg/mL; Fn—50 µg/mL) in a volume of 100 µL per cm^2^ well.

### 2.3. Microscope Evaluation

**Figure 3:**
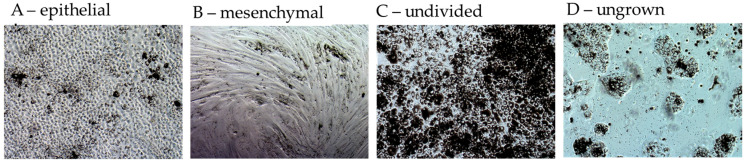
Example photographs of morphology. Morphological assessment was conducted with light microscopy images (5× magnification). Portions in % of the whole image of epithelial (**A**), mesenchymal (**B**), undivided (**C**) and ungrown cells (**D**) were visually determined by trained researchers.

Microscope Axiovert 160 with 5× objective was used to investigate the morphology of RPE cells after 7, 14 and 28 days of incubation. Imaging was performed with Axio Cam MRc5 and Axiovision Release 4.8.0.0 (Carl Zeiss AG) at three different positions of the well (5× magnification) with standardized coordinates (1. center, 2. middle ring, 3. edge of the well). Percent portion of epithelial, mesenchymal and undivided cells, and gaps between cells in one photo (summed up to 100%) were assessed by trained researchers according to a standardized photo evaluation protocol (including example images for the different RPE morphologies and percent ratios of the image; please refer to the example photos in Figure 3). As there were no relevant differences between the three well positions, the three replicates of data from one well were averaged. The evaluation was conducted blinded without knowledge about the experiment conditions. Also, day of full confluence (95–100% well area covered by cells) and portion of area (%) covered by cells after 7 days of cultivation were determined by trained researchers by a visual assessment of the cultures via light microscopy.

### 2.4. Enzyme-Linked Immunosorbent Assay

Supernatants of single-eye RPEs on Transwell inserts were collected after a cultivation time of 7, 14 and 28 days. Four hours before supernatant collection, the RPE media were renewed to ensure that the amount of secreted VEGF is within the upper detection range of the ELISA. Both, apical and basolateral supernatants were taken. They were centrifuged for 5 min at 16,000× *g*, 4 °C to remove cellular debris. VEGF content of supernatant was analyzed with Human VEGF DuoSet ELISA (R&D systems, Minneapolis, MN, USA; #DY293B), which is also capable of detecting porcine VEGF-A as it shows 100% protein sequence coverage (BLAST for proteins with human VEGF Isoform 165). ELISA was conducted as instructed by the manufacturer.

### 2.5. Fluorescence Imaging

For the assessment of cell morphometry and tight junctions, actin filaments, zonula occludens 1 (ZO-1) and cell nuclei were stained on cells cultured on Transwell inserts. The membrane was processed and stained for immunofluorescence (IF) as described below.

For assessing polarized bestrophin 1 expression (BEST1), cryosectioning (IHC-Fr) was performed prior to fluorescence staining. Here, optimal cutting temperature blocks (OCT blocks) were generated. For this, the media of wells were removed and wells were washed with warm PBS. Cells were fixated with 4% para-formaldehyde for 30 min. After the washing steps were conducted with PBS, the membrane of Transwell was cut out and cut into two halves. They were incubated in 10% saccharose solution (Carl Roth GmbH; #4661.1) for 30 min, 20% saccharose solution for 30 min and finally 30% saccharose solution overnight at 4 °C. The next day, membranes were put into OCT media Tissue-TEK (Sakura Finetek Germany GmbH, Umkirch, Germany; #4583) on ice to generate blocks. The blocks were attached on object tables (Thermo Fisher Scientific; #715610-CN) with Tissue-Tek Cryomold (Sakura Finetek Germany GmbH; #4557). Then, they were frozen and tightened in a Cryo Star Nx70 Cryostat (Thermo Fisher Scientific). As soon as membranes were cut (cutting thickness of 10 µm), they were collected on superfrost plus adhesion slides (Thermo Fisher Scientific; #J1800AMNZ).

For ZO-1 samples, samples were fixated in 4% para-formaldehyde (Carl Roth GmbH + Co. KG; #0335.1) and treated with 0.1% Triton-X (Carl Roth GmbH + Co. KG; #3051.2). IHC-Fr samples were fixated with 4% para-formaldehyde and washed with PBS plus Tween 20 (Sigma-Aldrich; #P9416). For all samples, BSA (3%) diluted in PBS was applied as a blocking reagent (Thermo Fisher Scientific; #37525). For ZO-1 tests, cells were treated for 1 h with rabbit ZO-1 antibody (Thermo Fisher Scientific; #61-7300, diluted 1:50); for BEST1 detection, it was stained with Anti-Bestrophin/BEST1 antibody [E6-6] (abcam, Cambridge, UK; #ab2182). After that, a staining solution consisting of Phalloidin-Atto488 (Sigma-Aldrich; #49409, diluted 1:50), Hoechst (Sigma-Aldrich; #14533, diluted 1:500) and Alexa Fluor 555 goat anti-rabbit (Thermo Fisher Scientific; #A32732, diluted 1:1000) or donkey anti-mouse (Thermo Fisher Scientific; #A31570, diluted 1:500) were applied for one hour. Membranes of the Transwell inserts (for ZO-1 and actin filaments staining) were cut out, treated with Fluoromount-G (Thermo Fisher Scientific; #00-4958-02) at the top of the microscope slides (Th. Geyer GmbH & Co. KG, Renningen, Germany; #42406010), and covered with glass slips (Th. Geyer GmbH & Co. KG, Renningen, Germany; #CB00180RA1). Slides for BEST1 detection were also mounted with Fluoromount-G and covered with glass slips.

**Figure 4:**
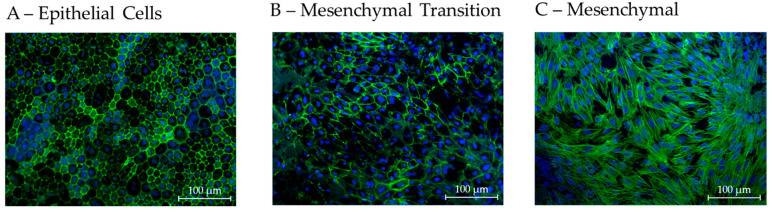
Example images for actin filament distribution as an indication of differentiation. To make a qualitative ranking of the overall RPE morphology, actin immune fluorescence imaging was used. (**A**) Epithelial honeycomb-structured cells depicting highly differentiated epithelial RPEs, (**B**) mixed cultures with a partial transformation to a mesenchymal phenotype with stress fibers and (**C**) completely mesenchymal cells.

Imager.M2 (Carl Zeiss AG) with 20× lens was used for fluorescence imaging of different positions of the membranes. ZO-1 expression and cell nuclei were used to determine cell morphometry using the CellProfiler Software (version 4.2.8; Broad Institute of MIT and Harvard, Cambridge, MA, USA). Photos with an area of 447.63 µm × 335.40 µm were used to determine cell number, cell area, form factor as well as perimeter, eccentricity and cell radius. Visually qualitatively best RPE photos from 28 days of cultivation (blinded for culture conditions) were used to determine cell parameters as the optimal polar single-eye RPE standard. For this, not only the ZO-1 channel but also the actin-filament channel was used to determine proper RPE morphology (Figure 4A), with a circumferential actin location beneath the cell membrane in epithelial, highly differentiated RPE cells. In contrast, lower-quality morphological RPEs showed disturbed actin filament and stress fibers (Figure 4B), or irregular actin filaments in completely mesenchymal cells (Figure 4C). For BEST1 expression and localization on either the apical or basolateral side of RPEs, Fiji (Image J2, version 2.9.0) was used to determine intensities and the area of the corresponding fluorescence signals.

### 2.6. Quantitative Polymerase Chain Reaction

To assess gene expression, RNA was isolated after a cultivation time of 28 days with three conditions (not coated, 10% serum; Lam, 5% serum; Lam, 10% serum). It was applied to a NulceoSpin RNA Mini Kit containing DNase to remove genomic DNA (Macherey-Nagel, Düren, Germany; #740955) as described by the manufacturer. RNA concentration and quality were analyzed with NanoDrop™ One (Thermo Fisher Scientific; #ND-ONE-W). cDNA was created with High-Capacity cDNA Reverse Transcription Kit (Thermo Fisher Scientific; #4368814), according to the instructions described by Thermo Fisher Scientific. Real-time relative and quantitative qPCR was conducted with TaqMan™ gene expression assays with dye label 5(6)-carboxyfluorescein-minor groove binder (FAM-MGB) (Thermo Fisher Scientific; #4351372) and TaqMan™ Fast Advanced Master Mix (Thermo Fisher Scientific; #4444557) as outlined in the manual of the master mix. Samples were applied on gene arrays for RPE-relevant genes (Section 3.3) according to Dörschmann et al. (2025) [16]. Endogenous controls for 18S-rRNA, *ACTG1*, *GAPDH* and *GUSB* were considered as candidate endogenous controls. For all data, CT values were transformed to ΔCT values using *ACTG1* expression [16,22] and these were used to calculate a relative quotient with the reference group co 10% (control with 10% serum but without coating).

### 2.7. Protein Expression

After 14 and 28 days of incubation, Claudin-19 (CLDN19) and retinoid isomerohydrolase (RPE65) protein expression were detected with Western blot. Samples were washed with PBS and treated with Nonidet P40 solution, with 1% Nonidet P40 Substitute (Sigma-Aldrich; #11332473001), 50 mM Tris (Sigma-Aldrich; #T1503, pH8) and 150 mM NaCl (Carl Roth GmbH + Co. KG; #3957.1) containing phosphatase inhibitor cocktail 1 and 2 (Sigma-Aldrich; #P2850, #P5726) and protease inhibitor cocktail (Sigma-Aldrich; #P8340) for 45 min. Proteins were determined with DC Protein Assay (Bio-Rad Laboratories, Munich, Germany; #5000111) as described in the manual. SDS-PAGE with 12% acrylamide gel and Western blot were conducted as described before with modifications [23]. PageRuler™ Plus Prestained Protein Ladder (Thermo Fisher Scientific; #26620), ranging from 10 to 250 kDa was used. Blocking was performed for 1 h with 4% skimmed milk (Carl Roth GmbH + Co. KG; #T145.2) in Tris buffered saline with 0.05% Tween (TBST, Merck KGaA, Darmstadt, Germany; #8221840). Primary antibodies were applied by gently shaking overnight at 4 °C (mouse anti-RPE65, 65 kDa, 1:2000 in 2% skimmed milk, Novus Biologicals, Minneapolis, MN, USA; #NB100-355; rabbit anti-CLDN19, 23 kDa, 1:1000 2% skimmed milk, Absource Diagnostics GmbH, München, Germany; #00219; rabbit anti-β-actin, 37 kDa, 1:1000 in 2% skimmed milk, Cell Signaling Technologies, Denver, CO, USA, #4967). Blots were washed with TBST and secondary conjugates were used for 1 h (horseradish peroxidase-conjugates, HRP, anti-mouse-HRP (Cell Signaling Technologies; #7076), anti-rabbit-HRP (Cell Signaling Technologies; #7074), both 1:1000 in 2% skimmed milk). Clarity Western ECL Substrate (Bio-Rad Laboratories; #170-5061) was added to the membranes for 5 min. Chemiluminescence signal was measured with ChemiDoc MP Imaging System (Bio-Rad Laboratories). Normalization was calculated with the expression of β-actin and bands’ volumes were determined with Image Lab 6.1.0 build 7 software (Bio-Rad Laboratories).

### 2.8. Statistical Analysis

The number of experiments corresponding to individual wells (single-eye cultures) used was at least three. Data managing, calculations and diagrams were performed with Microsoft Excel (Excel 2010, Microsoft, Redmond, WA, USA). Statistics were assessed with GraphPad Prism 10 (GraphPad Software, Inc., San Diego, CA, USA). Data distribution was assessed with Q-Q-Plot. Parametric data, depicted as bar graphs with mean and standard deviation, were evaluated first with ANOVA (analysis of variance), followed by Student’s *t*-test. Non-parametric data are depicted as whisker–boxplots with median, interquartile range and a range from minimum to maximum values. These data were evaluated first with Kruskal–Wallis, followed by the Mann–Whitney test. qPCR data were evaluated with Thermo Fisher Connect. Significance level was set to *p* = 0.05.

## 3. Results

### 3.1. Coating

#### 3.1.1. Cell Number Differences (Coatings)

**Figure 5:**
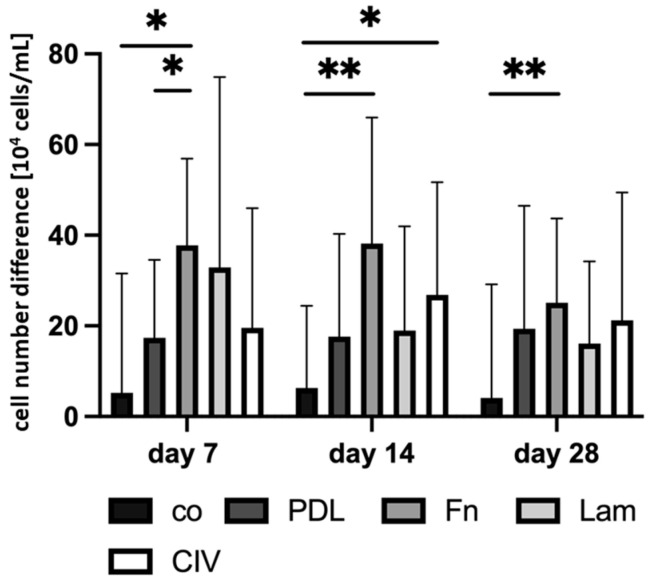
Cell number difference with different coatings. After a cultivation time of 7, 14 and 28 days, porcine single-eye retinal pigment epithelium cells were counted. They were seeded on non-coated wells (co), Poly-ᴅ-Lysine (PDL), fibronectin (Fn), laminin (Lam) or collagen IV (CIV). Change in cell number ×10^4^/mL at respective time points is shown. Data show normal distribution, mean and standard deviation. Between each condition, significances were determined via analysis of variance (ANOVA) and Student’s *t*-test. * *p* < 0.05, ** *p* < 0.01. *n* = 7–48.

Porcine single-eye RPE cells were cultivated on different plate coatings. In general, 10% serum was used for all coating experiments. Non-coated wells (co), as well as those with the coatings PDL, Fn, Lam, or CIV, were tested, and the cell numbers before and after seeding on days 7, 14 and 28 were determined. The overall seeding cell number for cultures used in this study was approximately 150,000 cells per well (14.97 ± 14.46 cells × 10^4^/mL). Cell number differences between the cell number at respective time points and seeding cell number are shown in Figure 5. For all time points, Fn showed the highest cell number difference. After 7 days, Fn had significantly more cells compared to co (*p* = 0.018) and PDL (*p* = 0.025). After 14 days, Fn’s cell number difference was significantly higher than that of co (*p* = 0.003); CIV also showed a significantly higher cell number difference than co (*p* = 0.025). After 28 days, again, Fn coating resulted in a higher cell number difference than co (*p* = 0.005). Overall, Fn showed the highest cell number growth whereas co had the lowest.

#### 3.1.2. Confluence (Coatings)

**Figure 6:**
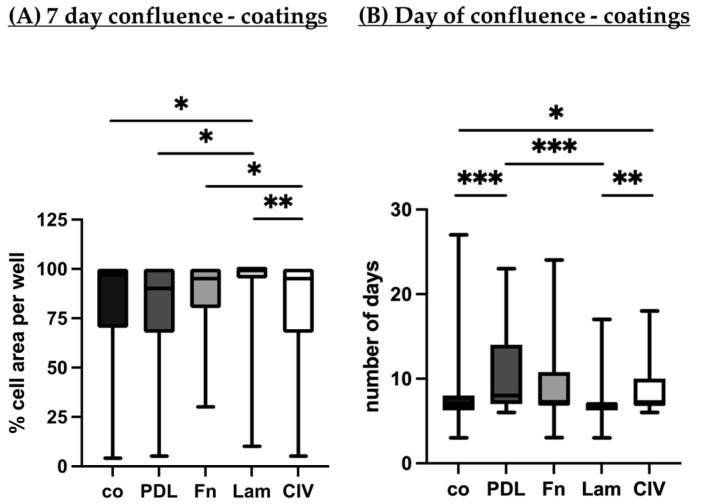
Confluence with different coatings. Porcine single-eye retinal pigment epithelium cells were cultured on non-coated wells (co), Poly-ᴅ-Lysine (PDL), fibronectin (Fn), laminin (Lam), or collagen IV (CIV). On the 7th day of cultivation, confluent cell growth area was determined by light microscopy in % of the whole well (**A**). In addition, day of full confluence was determined (**B**). Data are non-parametric; median, interquartile range and range from minimum to maximum are depicted. Between each group, significances were calculated with the Kruskal–Wallis test followed by Mann–Whitney test. * *p* < 0.05, ** *p* < 0.01, *** *p* < 0.001. *n* = 45–89.

Porcine single-eye RPE cells were cultured on Transwell inserts with different coatings (co, PDL, Fn, Lam, or CIV). Using light microscopy photos, cell growth area on day 7 (in %) of the whole well (Figure 6A) and the day of full confluence (Figure 6B) were determined. Regarding the 7-day-confluence, Lam showed the highest cell growth area, which was significantly higher than co (*p* = 0.045), PDL (*p* = 0.022) and CIV (*p* = 0.009). Conversely, CIV had a growth area with a significantly lower range than Fn (*p* = 0.032). Concerning day of full confluence, Lam was significantly more efficient than PDL (*p* < 0.001) and CIV (*p* = 0.004). Also, co showed more efficient confluence results compared to PDL (*p* = 0.001) and CIV (*p* = 0.020). Taken together, Lam exhibited the most efficient cell growth and confluence activities. Of note, this is not a contradiction to the higher cell number seen with fibronectin (Section 3.1.1), as the coverage of a specific area is dependent not only on cell number, but on cell shape. A hexagonal shape, as found in differentiated RPE cells, is optimized for the most efficient coverage of a given area, minimizing the number of cells needed [24,25].

#### 3.1.3. Morphology (Coatings)

**Figure 7:**
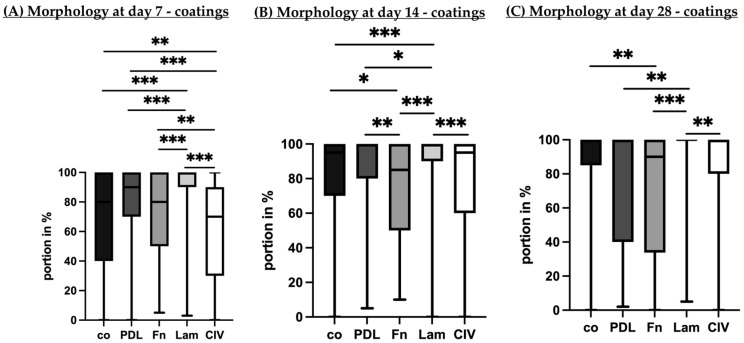
Cell morphology on different coatings. Porcine single-eye retinal pigment epithelium cells were cultivated on non-coated wells (co), Poly-ᴅ-Lysine (PDL), fibronectin (Fn), laminin (Lam) or collagen IV (CIV). After 7 (**A**), 14 (**B**) and 28 (**C**) days, the portion of epithelial areas of the individual wells was analyzed with bright field imaging. Data are non-parametric; median, interquartile range and range from minimum to maximum are depicted. Between each group, significances were calculated with Kruskal–Wallis test followed by Mann–Whitney test. * *p* < 0.05, ** *p* < 0.01, *** *p* < 0.001. *n* = 18–86.

Porcine single-eye RPEs were cultivated on Transwell inserts with different coatings (co, PDL, Fn, Lam, or CIV) for 7 (Figure 7A), 14 (Figure 7B) and 28 (Figure 7C) days. Bright field microscope imaging of the same individual well was conducted to determine the portion in percent of epithelial cells, mesenchymal cells, undivided cells and cell layer gaps. The entire morphological assessment is depicted in Supplementary Materials (Figure S1). Here, our focus is on epithelial morphology.

After seven days of cultivation, Lam showed the highest portion of epithelial cells, which was significantly higher than co (*p* < 0.001), PDL (*p* = 0.002), Fn (*p* < 0.001) or CIV (*p* < 0.001). Conversely, CIV had significantly lesser epithelial cells than Fn (*p* = 0.003), PDL (*p* < 0.001) or co (*p* = 0.005). After 14 days of cultivation, Lam once again showed the highest portion of epithelial cells. Also, Fn had lesser epithelial cells than PDL (*p* = 0.008) and co (*p* = 0.034). After 28 days of cultivation, Lam showed complete epithelial morphology, with other conditions showing more similar results but higher variation. Also, co had more epithelial cells than Fn (*p* = 0.003). Altogether, Lam showed the best epithelial morphological differentiation at all time points assessed, while other morphology parameters were low. Descriptively, Lam showed the lowest portion of mesenchymal cells, undivided cells and ungrown areas on day 7, which corresponds to the efficient confluence activities of Section 3.1.2 (Figure S1). On day 28, Lam had the lowest portion of undivided cells and ungrown areas as well (refer Figure S1). Fn, in contrast, showed high portions of mesenchymal and undivided cells as well as ungrown areas for all time points (Figure S1).

#### 3.1.4. Success Rates (Coatings)

For all the porcine single-eye RPE cultures used for coating tests in Section 3.1, culture statistics and success rates were calculated. In Table 1, living culture rates (survived cultures/seeded cultures) and confluence culture rates (confluent cultures/survived cultures) are listed. For absolute data and culture counts, please refer to Table S1 in Supplementary Materials.

Altogether, 376 eyes were prepared, from which 71 RPE cultures did not survive for the indicated cultivation time, which makes a success rate of 81% for the preparation of single-eye RPEs on Transwell inserts (Table S1). From the surviving cultures, 252 reached confluence, which makes a confluence success rate of 83% for surviving polar single-eye RPE cultures (Table S1). Concerning the coating substance, Lam and Fn showed the best success rates, whereas co and PDL showed the worst. All cultures on Lam- and Fn-coated wells were alive after seven days of cultivation, whereas co exhibited a lower survival rate. Also, Lam had the highest confluence rate, in contrast to PDL having the lowest confluence rate. After 14 days, Fn still showed a complete survival rate, which was the highest among the coatings, whereas PDL was the lowest, but Lam and CIV were also highly effective. Concerning confluence rate, Lam showed the highest and co the lowest. After 28 days, Fn still had the highest survival rate whereas co was the lowest. Lam had the highest confluence success rate and co the worst. Altogether, the coating of Transwell inserts is recommended due to the low success rates for non-coated wells and Lam is recommended as it has high success rates in general. Fn was also efficient concerning survival rates, but the results in the previous section argue against using Fn as a coating as it shows poor differentiation in epithelioid phenotypes.

#### 3.1.5. Angiogenic Factor Secretion (Coatings)

**Figure 8:**
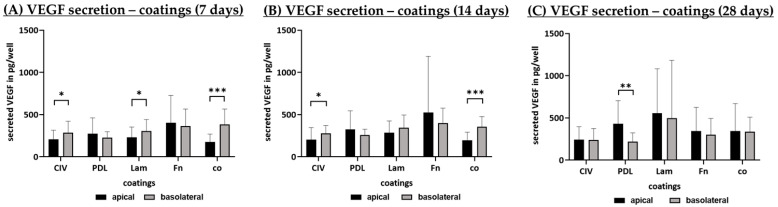
VEGF secretion with different coatings. Porcine single-eye retinal pigment epithelium cells were cultivated on Transwell inserts coated with collagen IV (CIV), laminin (Lam), Poly-ᴅ-Lysine (PDL), fibronectin (Fn) or non-coated wells (co) for 7 (**A**), 14 (**B**) and 28 (**C**) days. Apical and basolateral supernatants were harvested for four hours at different cultivation times and applied in ELISA to determine vascular endothelial growth factor A (VEGF) secretion in pg per well area. Data were parametric; mean and standard deviation are depicted. Between each group, significances were first calculated with analysis of variance (ANOVA) followed by Student’s *t*-test. * *p* < 0.05, ** *p* < 0.01, *** *p* < 0.001. *n* = 14–19.

Secreted VEGF of polar single-eye RPE cultures on Transwell inserts coated with CIV, PDL, Lam, Fn or co was determined with ELISA. Cells were incubated for 7 (Figure 8A), 14 (Figure 8B) and 28 (Figure 8C) days and apical as well as basolateral supernatants were collected for 4 h. VEGF secretion is displayed in pg per well area. Lam coating showed the strongest VEGF secretion activity after 28 days of cultivation. In general, after 28 days, apical VEGF secretion ranged between 244.36 ± 152.27 pg/mL (CIV) and 558.63 ± 524.36 pg/mL (Lam) and basolateral secretion ranged from 221.56 ± 102.59 pg/mL (PDL) to 499.60 ± 682.89 pg/mL (Lam), which, in both cases, is lower than VEGF secretion in non-polar 12-well plates with 735.33 to 1109.33 pg per well [16]. Concerning polarity of VEGF secretion, in co after 7 days, basolateral secretion was significantly higher than apical (*p* < 0.001), which could be reproduced after 14 days (*p* < 0.001) but not after 28 days. PDL apical secretion was significantly higher than basolateral secretion after 28 days (*p* = 0.006) of cultivation. After 7 and 14 days, CIV basolateral secretion was significantly higher than apical secretion (*p* = 0.036, *p* = 0.017, respectively), which was also the case for Lam after 7 days (*p* = 0.014).

#### 3.1.6. Protein Expression (Coatings)

**Figure 9:**
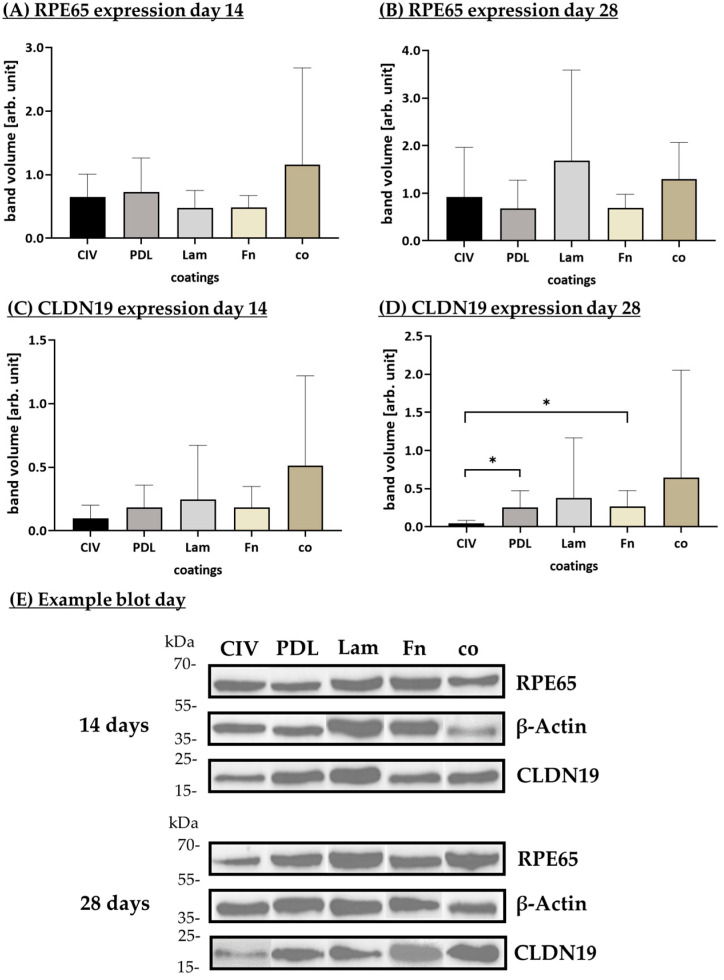
CLDN19 and RPE65 expression with different coatings. Porcine single-eye retinal pigment epithelium cells were cultivated on Transwell inserts coated with collagen IV (CIV), laminin (Lam), Poly-ᴅ-Lysine (PDL), fibronectin (Fn), or non-coated wells (co) for 14 (**A**,**C**) and 28 (**B**,**D**) days. Lysates were analyzed with Western blot for retinoid isomerohydrolase (RPE65, (**A**,**B**)) and claudin-19 (CLDN19, (**C**,**D**)). Example blots are shown (**E**). Data were parametric; mean and standard deviation are depicted. Between each group, significances were first calculated with analysis of variance (ANOVA) followed by Student’s *t*-test. * *p* < 0.05. *n* = 6–7.

CLDN19 and RPE65 are proteins expressed by differentiated RPE cells and are indicative of proper tight junctions and visual recycling, respectively [2]. To examine their expression, porcine single-eye RPE were seeded on Transwell inserts coated with CIV, PDL, Lam, Fn or co. Protein expression was assessed with Western blot after 14 and 28 days (example blots in Figure 9E) of cultivation.

Concerning the 14th day, CLDN19 expression (Figure 9A) was increased with no-coating control co and decreased with CIV coating, but no significance could be found. RPE65 expression (Figure 9C) was increased with co and lowered with Lam, but no significances were reached. After 28 days of cultivation, the expressed CLDN19 (Figure 9B) was, again, highest with co and lowest with CIV, which was significantly lower than CLDN19 expression on PDL (*p* = 0.049) and Fn (*p* = 0.031). RPE65 expression was highest with Lam coating and lowest with PDL coating, with no significant findings. Overall, the expression of the assessed proteins varied between the wells, indicating that the origin of the RPE cells had a stronger influence on its quantitative expression than the coatings used, with the exception of CIV, which seem to result in a lower expression of CLDN19.

#### 3.1.7. Cell Barrier (Coatings)

**Figure 10:**
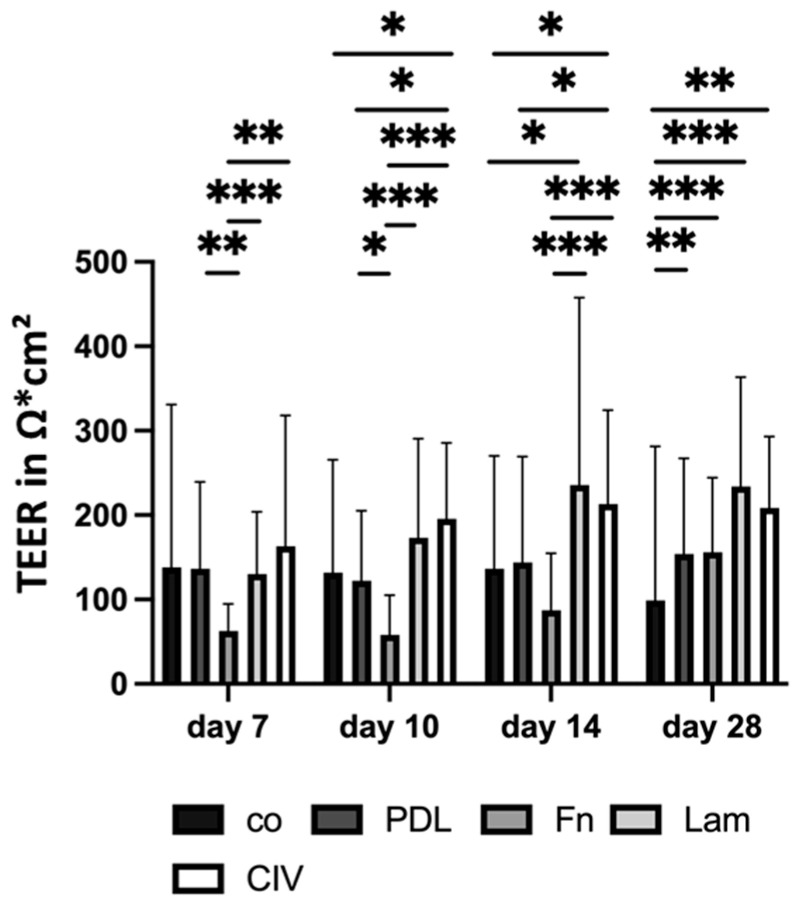
Cell barrier with different coatings. Porcine single-eye retinal pigment epithelium cells were cultivated on Transwell inserts non-coated (co) or coated with Poly-ᴅ-Lysine (PDL), fibronectin (Fn), laminin (Lam) or collagen IV (CIV) for 7, 10, 14 and 28 days. Transepithelial electrical resistance (TEER) was measured. Data were parametric; mean and standard deviation are depicted. Between each group, significances were calculated with analysis of variance (ANOVA) followed by Student’s *t*-test. * *p* < 0.05, ** *p* < 0.01, *** *p* < 0.001. *n* = 9–62.

Proper differentiated RPE cells that form a tight cell barrier uphold the outer blood–retina barrier, which can be assessed on Transwell plates by TEER. For this, primary porcine single-eye RPEs were cultured on coated Transwell inserts for 28 days, and TEER was measured in the same individual wells on days 7, 10, 14 and 28 (Figure 10). Co, PDL, Fn, Lam and CIV were tested. In general, from day 10 onwards, CIV and Lam showed the highest TEER values. On day 7, Fn coating showed the lowest TEER measurements (mean: 62.91 ± 31.72 Ω*cm^2^), which was significantly lower than PDL (*p* = 0.001), Lam (*p* < 0.001) or CIV (*p* = 0.001). On day 10, CIV displayed the highest TEER values (mean: 195.52 ± 90.19 Ω*cm^2^), which was significantly higher than co (*p* = 0.015), PDL (*p* = 0.013), or Fn (*p* < 0.001). Fn was also significantly lower than PDL (*p* = 0.028) and Lam (*p* < 0.001). From day 14 onwards, Lam showed the highest TEER (mean: 235.51 ± 222.04 Ω*cm^2^) and this was significantly higher than co (*p* = 0.014) or Fn (*p* < 0.001). In addition, CIV showed a significantly higher TEER than co (*p* = 0.010), PDL (*p* = 0.023), or Fn (*p* < 0.001). On day 28, co displayed the lowest TEER value (99.14 ± 182.16 Ω*cm^2^), which was significantly different to PDL (*p* = 0.008), Fn (*p* = 0.001), Lam (mean: 233.86 ± 129.49 Ω*cm^2^, *p* < 0.001) and CIV (*p* = 0.001). Overall, the coating of Transwell inserts is important for the formation of a right cell barrier and Lam showed the best results, starting after two weeks of cultivation.

#### 3.1.8. Cell Parameters (Coatings)

**Figure 11:**
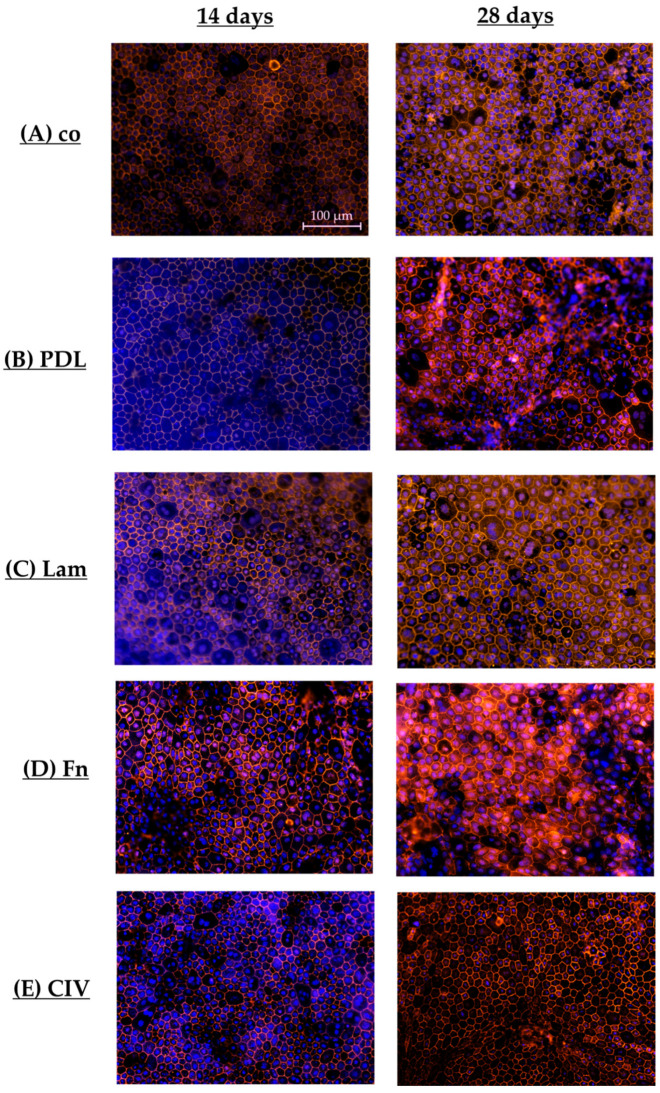
Exemplary photos. Cells used for determining morphometric parameters were stained for cell nuclei and tight junctions. Example photos for each coating condition are shown 14 and 28 days after preparation (non-coated wells (co, (**A**)), Poly-ᴅ-Lysine (PDL, (**B**)), laminin (Lam, (**C**)), fibronectin (Fn, (**D**)), or collagen IV (CIV, (**E**)). Objective = 20×; scale bar = 100 µm.

To examine polar single-eye RPE tight junctions and cell parameters, RPE cells were seeded on Transwell inserts non-coated (co) or coated with PDL, Lam, Fn or CIV, for 14 or 28 days. After that, Transwell membranes were stained for cell nuclei and tight junctions’ protein ZO-1, taking fluorescence photos (example photos shown in Figure 11). These were evaluated with the CellProfiler Software. RPE morphometric standard values were determined in a former study [16]. By visually ranking the IF photos from 28 days of cultivation and choosing the best RPE morphology fitting pictures for the CellProfiler evaluation, optimal cell parameters for polar single-eye RPEs were determined. In Table 2 and Table 3, the most important parameters are listed. For all parametric data, please refer to Tables S2 and S3 in Supplementary Materials.

In general, polar and non-polar [16] RPE standards differ. The cell number on Transwell plates is higher compared to non-polar plates (529.88 cells (polar) vs. 472.31 cells (non-polar)), and the cell area is smaller (267.05 µm^2^ (polar) vs. 324.25 µm^2^ (non-polar)). Form factor for polar RPEs is smaller (0.59 (polar) vs. 0.68 (non-polar)). Taken together, optimal single-eye RPEs on Transwell inserts are higher in cell number and smaller in cell shape, and both models do not show an equilateral hexagonal value (form factor 0.84), which can usually be found in the macula of humans [26], showing pentagonal- or hexagonal-shaped cells with uneven cell sites.

According to different culture conditions, notably, after 14 days of cultivation (Table 2), the mean metric values of all conditions differ considerably from the calculated standard for polar RPE single-eye cultures, strongly indicating that the cells still grow and mature (e.g., polar standard cell area 267.05 µm^2^ (28 d photos) compared to polar mean with 201.85 µm^2^ (14 days photos)). Mean cell number for Transwell was higher than the polar RPE standard. In general, mean form factor was higher than the polar standard. Using Lam showed the closest result to the polar standard concerning cell number and the overall lowest value among the coatings, whereas co showed the highest values. Cell area was the largest when using Lam, which was also the closest to the polar, while co conversely showed the smallest area. The form factor of all coatings was also similar compared to the non-polar standard.

After 28 days of cultivation (Table 3), polar mean data were closer to the polar RPE standard, while the cell areas and numbers changed remarkably (mean: 272.88 µm^2^ compared to 267.05 µm^2^ of the polar standard; mean: 507.87 cells compared to 529.88 cells of the standard). Form factor was comparable.

Concerning cell number, PDL showed the highest, whereas Lam showed the lowest. CIV was closest to the polar standard. Also, compared to the polar standard, Lam showed, again, the largest cell area but was the least close to the standard, whereas PDL showed the lowest cell area. All conditions, and especially Lam, had a lower form factor compared to day 14, which showed more pentagonal- or hexagonal-shaped cells with uneven cell sites. CIV had the largest form factor and PDL as well as Fn were close to the standard. Overall, polar RPE single-eye cells differ from non-polar RPE cells in morphometry. Furthermore, our data show that the cell morphometry, especially number and cell size, differ strongly between 14 and 28 days of cultivation. Lam showed a low cell number but high cell metric values. As shown in Figure 11C, polar RPEs on Lam showed bigger cells compared to other coatings, but similar cell sizes to the non-polar standard. Also, Lam showed non-homogeneous cell morphometrics with cell site number changing between 5 and 7 (which are also uneven), which explains the lower form factor. Other coatings showed smaller, more compact, and evenly shaped cells (Figure 11A,B,D,E).

#### 3.1.9. Protein Localization (Coatings)

**Figure 12:**
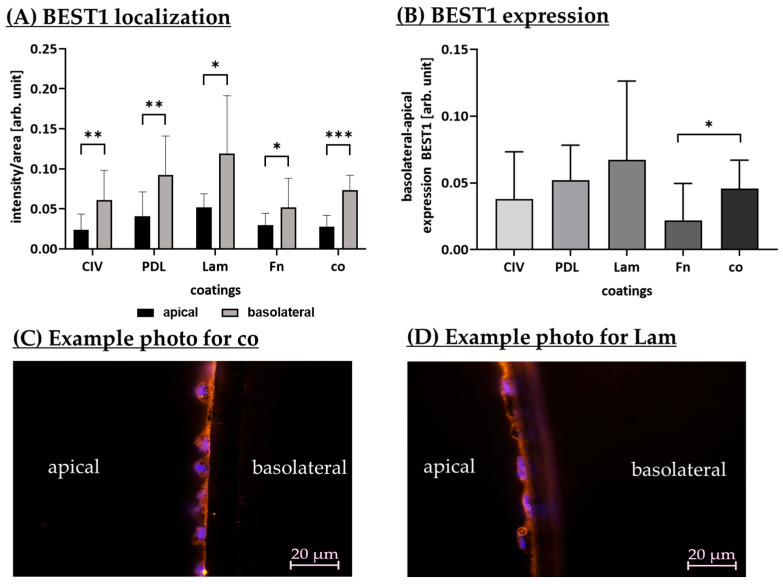
Bestrophin-1 localization and expression. Porcine single-eye retinal pigment epithelium cells were cultivated on Transwell inserts coated with collagen IV (CIV), laminin (Lam), Poly-ᴅ-Lysine (PDL), fibronectin (Fn), or non-coated wells (co) for 28 days. Cryosectioning of Transwell inserts was performed and stained for bestrophin-1 (BEST1) expression. Example photos for co (**C**) and Lam (**D**) are depicted (green = cell nuclei, orange = BEST1, objective = 63×; scale bar = 20 µm). Apical and basolateral expression was detected and evaluated with Fiji. Apical and basolateral fluorescence intensity normalized by fluorescence area (**A**) as well as relative basolateral and apical normalized fluorescence intensity (**B**) are shown. Data were parametric; mean and standard deviation are depicted. Between each group, significances were first calculated with analysis of variance (ANOVA) followed by Student’s *t*-test. * *p* < 0.05, ** *p* < 0.01, *** *p* < 0.001. *n* = 6–14.

Different coatings may have an influence on the polar protein expression of RPEs. To investigate this, polar porcine single-eye retinal pigment epithelium cells were cultivated on Transwell inserts coated with CIV, PDL, Lam or Fn, or using non-coated wells (co) for 28 days. Cryosectioning of Transwell inserts was performed and they were stained for RPE differentiation marker bestrophin-1 (BEST1), which is physiologically higher expressed on the basolateral RPE side. Apical and basolateral expression was detected and evaluated with Fiji. Apical and basolateral fluorescence intensity normalized by fluorescence area (Figure 12A) as well as relative basolateral and apical normalized fluorescence intensity (Figure 12B) are shown.

Concerning localization of BEST1, for all coating conditions and co, basolateral BEST1 expression significantly exceeded apical expression as expected for differentiated RPE cells. For CIV, PDL, Lam, Fn and co, basolateral BEST1 had a higher intensity compared to its apical expression (*p* = 0.005, *p* = 0.005, *p* = 0.024, *p* = 0.045, and *p* < 0.001, respectively).

Concerning relative basolateral BEST1 expression, Lam showed the highest basolateral protein signal, whereas Fn had the lowest one, which was significantly different compared to co (*p* = 0.029). Exemplary polar BEST1 expression is shown for standard co (Figure 12C) and the best condition, Lam (Figure 12D). Taken together, Lam seems to be a suitable coating concerning polar RPE differentiation.

### 3.2. Serum Content

Based on the results obtained in Section 3.2. (serum content tests) Lam-coated Transwell cultures were used compared to co (non-coated wells) as Lam proved to be the best coating substance.

#### 3.2.1. Cell Number Differences (Serum Content)

**Figure 13:**
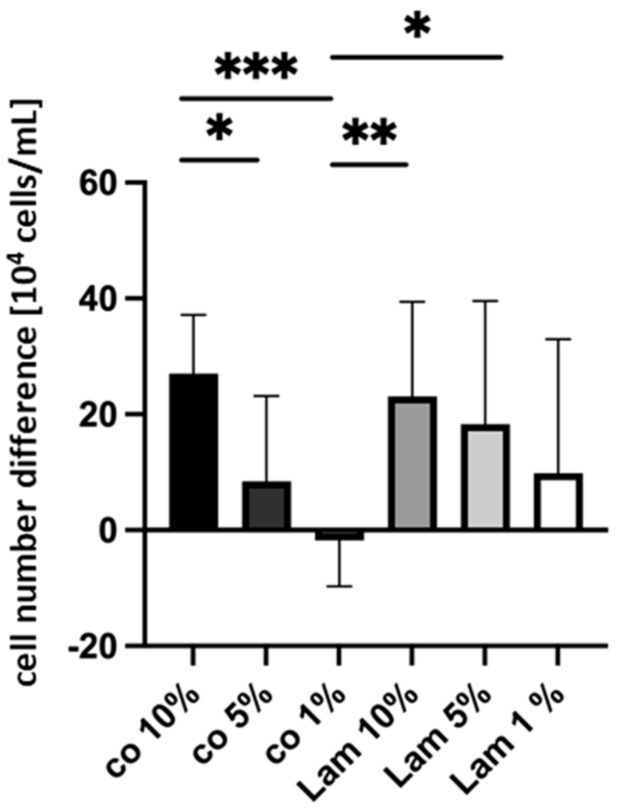
Cell number difference depending on serum content. Porcine single-eye retinal pigment epithelium cells were counted with trypan-blue exclusion assay after 14 days of cultivation. They were seeded on laminin (Lam) or non-coated wells (co) with different serum content (1%, 5%, 10%). Cell number difference x10^4^/mL is shown. Data show normal distribution; mean and standard deviation are depicted. Between each condition, significances were determined via analysis of variance (ANOVA) and Student’s *t*-test. * *p* < 0.05, ** *p* < 0.01, *** *p* < 0.001. *n* = 5–7.

Porcine single-eye cells from RPEs were cultivated on Lam or non-coated wells (co) with different serum contents of 1%, 5% or 10%, and cell numbers before seeding and after 14 days were determined. Fourteen days were selected as the most meaningful time point for cell number differences, as cell numbers at 7 days display high variation, and after 28 days, are all similar due to full cell confluence. Cell number differences are shown in Figure 13. Cell number differences increased with higher serum content; cell number difference with co 1% was negative, which was significantly lower than co 10% (*p* < 0.001), Lam 10% (*p* = 0.005), or Lam 5% (*p* = 0.043). Also, co 10% showed a significantly higher cell number increase than co 5% (*p* = 0.042). A higher serum content results in higher cell number growth.

#### 3.2.2. Confluence (Serum Content)

**Figure 14:**
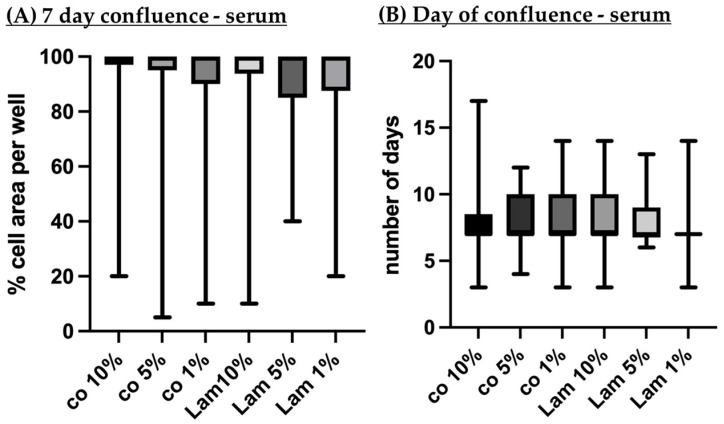
Confluence depending on serum content. Porcine single-eye retinal pigment epithelium cells were cultured on laminin (Lam), or non-coated wells (co) with different serum content (1%, 5%, 10%). On the seventh day of cultivation, confluent cell growth area was determined by bright field microscopy in % of the well (**A**). Also, day of full confluence was determined (**B**). Data are non-parametric; median, interquartile range and range from minimum to maximum are depicted. Between each group, significances were calculated with Kruskal–Wallis test followed by Mann–Whitney test. No significant findings were found. *n* = 22–40.

Porcine single-eye RPE cells were cultured on Transwell inserts coated with Lam or non-coated (co). With light microscope photos, cell growth area on day 7 in % of the whole well (Figure 14A) and day of full confluence (Figure 14B) were determined. There were no significant differences found. All conditions showed a median of 100% cell growth area on day 7. Also, all conditions reach full confluence after a median of 7 days.

#### 3.2.3. Morphology (Serum Content)

**Figure 15:**
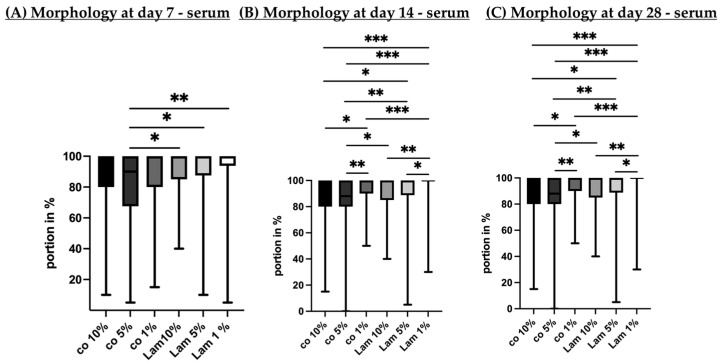
Cell morphology depending on serum content. Porcine single-eye retinal pigment epithelium cells were cultured on laminin (Lam) or non-coated wells (co) with different serum contents (1%, 5%, 10%). After 7 (**A**), 14 (**B**) and 28 (**C**) days, the portion of epithelial areas of the individual wells was determined with bright field microscopy photos. Data are non-parametric; median, interquartile range and range from minimum to maximum are depicted. Between each group, significances were calculated with Kruskal–Wallis test followed by Mann–Whitney test. * *p* < 0.05, ** *p* < 0.01, *** *p* < 0.001. *n* = 48–54.

Porcine single-eye RPE cells were cultivated on Transwell inserts coated with Lam or non-coated ones with different serum contents (1%, 5%, 10%) for 7 (Figure 15A), 14 (Figure 15B) and 28 (Figure 15C) days. Light microscope imaging of the individual wells was conducted to determine the portion in percent of epithelial cells, mesenchymal cells and undivided cells plus gaps between cells. The whole morphological assessment is depicted in Supplementary Materials (Figure S2). Here, our focus is on epithelial morphology.

After 7 days of cultivation, Lam 10% showed the highest portion of epithelial cells and co 5% had the lowest amount, which was significantly lower compared to Lam 10% (*p* = 0.039), Lam 5% (*p* = 0.014) and Lam 1% (*p* = 0.004). After 14 days of cultivation, epithelial cell portion was at a maximum with Lam 1%, whereas co 5% had the lowest one (*p* < 0.001) compared to Lam 1%. After 28 days, Lam 10% had significantly higher varying epithelial cell formation than co 5% (*p* = 0.011), Lam 5% (*p* = 0.025) and Lam 1% (*p* = 0.003). Also, co 5% had significantly less epithelial cells than co 10% (*p* = 0.001) and co 1% (*p* = 0.012). Of note, Lam 1% shows the least mesenchymal cell formation, and from day 14 onwards, it shows the least undivided cells (Table S2). In general, the lower the serum content on Lam-coated wells, the higher the proper RPE cell morphology. On day 28, Lam 1% and Lam 5% showed similar results.

#### 3.2.4. Success Rates (Serum Content)

For all the porcine single-eye RPE cultures used for serum tests in Section 3.2, culture statistics and success rates were calculated. In Table 4, living culture rates (survived cultures/seeded cultures) and confluence culture rates (confluent cultures/survived cultures) are listed. For absolute data and culture counts, please refer to Table S4 in Supplementary Materials.

Altogether, 205 porcine eyes were prepared, from which 17 RPE cultures did not survive for the indicated cultivation time, which makes a success rate of 91%, which is 10% higher than the success rate for coating tests (Section 3.1.4), thus exhibiting the benefits of Lam as a coating (Table S4). From the living cultures, 167 exhibited complete confluency, which makes a confluence success rate of 88%, which is 5% higher than the coating tests (Section 3.1.4, Table S4). According to the serum condition used, survival rate for all conditions was 100% after 7 days of cultivation, whereas Lam 1% had the highest confluence success, and co 1% and Lam 5% showed the lowest but still comparable confluence rate. After 14 days, co 1% and Lam 5% still had a 100% survival rate (comparable with Lam 1%) while co 10% exhibited the lowest survival rate. On the other hand, co 10% and Lam 10% had a 100% confluence rate, whereas Lam 5% and Lam 1% were the lowest but still efficient. On day 28, Lam 5% was the only condition with a 100% survival rate followed by co 5%, co 1% and Lam 1%, while co 10% showed lowest survival rate. But, again, co 10% and Lam 10% had a 100% confluence rate, whereas the other four conditions with lowered sera were still high. Altogether, a serum content lower than 10% leads to better cell culture success in long-term cultivation, but confluence success is slightly better with higher serum content, irrespective of whether Lam is used as a coating.

#### 3.2.5. Angiogenic Factor Secretion (Serum Content)

**Figure 16:**
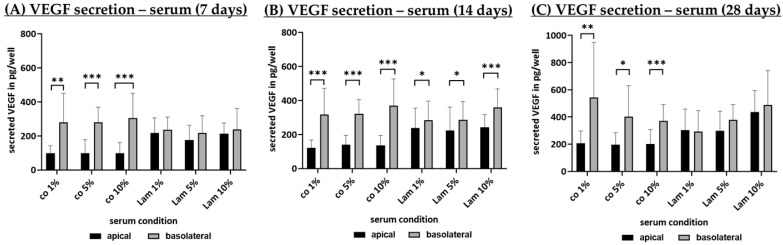
VEGF secretion depending on serum content. Porcine single-eye retinal pigment epithelium cells were cultured in laminin (Lam), or non-coated Transwell inserts (co) with different serum contents (1%, 5%, 10%) for 7 (**A**), 14 (**B**) and 28 (**C**) days. Apical and basolateral supernatants were harvested for four hours at the respective cultivation time and applied in ELISA to determine vascular endothelial growth factor A (VEGF) secretion in pg per well area. Data are parametric; mean and standard deviation are depicted. Between each group, significances were first calculated with analysis of variance (ANOVA) followed by Student’s *t*-test. * *p* < 0.05, ** *p* < 0.01, *** *p* < 0.001. *n* = 10–14.

VEGF secretion of polar single-eye RPE cultures on Transwell inserts coated with Lam or non-coated (co), treated with different portions of serum (1%, 5%, 10%), was determined with ELISA. Cells were incubated for 7 (Figure 16A), 14 (Figure 16B) and 28 (Figure 16C) days and apical as well as basolateral supernatants were collected after 4 h. After 28 days of cultivation, apical VEGF secretion ranged from 196.29 ± 89.00 pg/mL (co 5%) to 437.16 ± 158.35 pg/mL (Lam 10%), whereas basolateral secretion ranged from 293.92 ± 153.26 pg/mL (Lam 1%) to 542.57 ± 406.33 pg/mL (co 1%). For all time points, basolateral VEGF secretion was descriptively higher, independent of the serum content, with the exception of Lam 1% on day 28. Also, Lam shows a general higher apical secretion than co. After 7 days, co 1%, co 5% and co 10% showed significantly higher VEGF secretion on the basolateral side compared to the apical (*p* = 0.003, *p* < 0.001, *p* < 0.001, respectively), while Lam showed no significant differences. After 14 days, all six conditions showed significantly higher VEGF secretion on the basolateral side compared to apical (*p* < 0.001 to *p* = 0.04). After 28 days, co 1%, co 5% and co 10% still showed significantly higher basolateral secretion (*p* = 0.005, *p* = 0.011, *p* < 0.001, respectively), which was not significant for Lam.

#### 3.2.6. Protein Expression (Serum Content)

**Figure 17:**
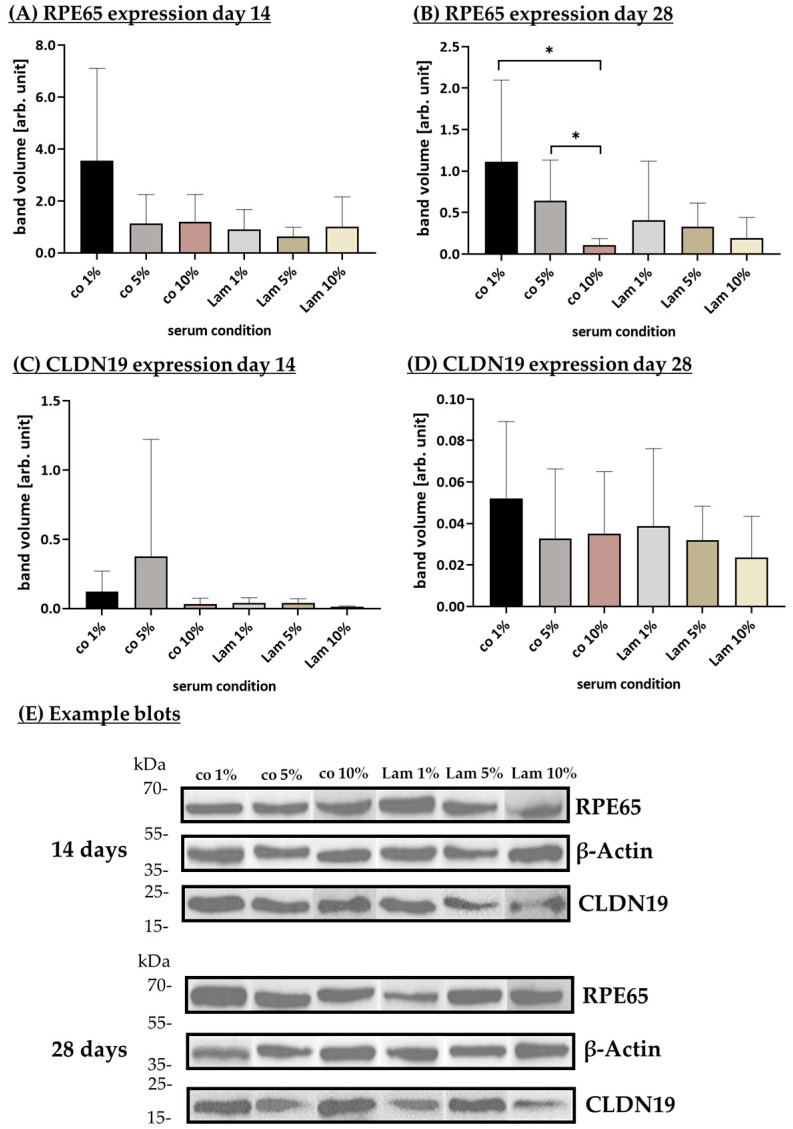
RPE65 and CLDN19 expression with serum content. Porcine single-eye retinal pigment epithelium cells were cultured in laminin (Lam) or non-coated Transwell inserts (co) with different serum contents (1%, 5%, 10%) for 14 (**A**,**C**) and 28 (**B**,**D**) days. Lysates were analyzed with Western blot for retinoid isomerohydrolase (RPE65, (**A**,**B**)) and claudin-19 (CLDN19, (**C**,**D**)). Example blots are shown (**E**). Data are parametric; mean and standard deviation are depicted. Between each group, significances were first calculated with analysis of variance (ANOVA) followed by Student’s *t*-test. * *p* < 0.05. *n* = 6.

To examine CLDN19 and RPE65 protein expression with different portions of serum, porcine single-eye RPEs were seeded on Transwell inserts coated with Lam or non-coated (co), treated with 1%, 5% or 10% serum content. Protein expression was assessed with Western blot after 14 and 28 days (example blots in Figure 17E) of cultivation.

Concerning 14 days, CLDN19 expression (Figure 17A) was not significantly different between conditions but, descriptively, Lam 10% showed the lowest expression and co 5% showed the highest expression as well as high deviation. Also, for RPE65 expression (Figure 17C), there was no significant difference, but Lam 5% showed the lowest expression in contrast to co 1%, with the highest expression but also the highest deviation. Regarding 28 days, CLDN19 expression (Figure 17B) showed, again, no significant differences, but this time, co 1% showed the highest expression in contrast to Lam 10% with the lowest one. RPE expression (Figure 17D) was least expressed with co 10% while co 5% (*p* = 0.025) and co 1% (*p* = 0.032) showed significantly higher protein expression with the latter also being the highest one. Taken together, the reduction in serum seems to be positive for RPE protein expression on day 28.

#### 3.2.7. Cell Barrier (Serum Content)

**Figure 18:**
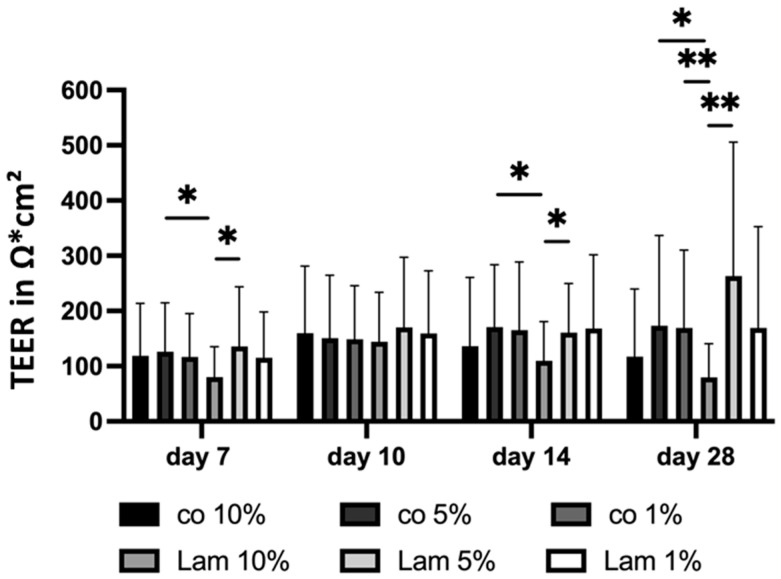
Cell barrier depending on serum content. Porcine single-eye retinal pigment epithelium cells were cultured on Transwell inserts coated with laminin (Lam), or non-coated Transwell inserts (co) with different serum contents (1%, 5%, 10%) for 7, 10, 14, and 28 days. Transepithelial electrical resistance (TEER) was measured. Data are parametric; mean and standard deviation are depicted. Significances were calculated with analysis of variance (ANOVA) followed by Student’s *t*-test. * *p* < 0.05, ** *p* < 0.01. *n* = 9–62.

To assess cell barrier properties depending on the serum content of the media, primary porcine single-eye RPE cells were cultured on Transwell inserts coated with Lam or non-coated (co) with different serum contents (1%, 5%, 10%) for 28 days, and TEER was measured in the individual wells on day 7, 10, 14 and 28 (Figure 18). At day 7, Lam 10% showed the lowest barrier (mean: 80.21 ± 55.26 Ω*cm^2^), with co 5% (*p* = 0.023) and Lam 5% (*p* = 0.045) displaying significantly higher TEER. Lam 1% also had a proper barrier (mean: 114.30 ± 83.77 Ω*cm^2^). At day 10, there were no significant or relevant differences. At day 14, Lam 10% had the lowest TEER values (mean: 109.52 ± 71.10 Ω*cm^2^) with co 5% (*p* = 0.015), Lam 5% (*p* = 0.018) displayed a significantly higher barrier, and Lam 1% showed a proper barrier (mean: 167.50 ± 133.67 Ω*cm^2^). After 28 days of cultivation, again, Lam 10% showed the lowest cell barrier (mean: 79.78 ± 61.14 Ω*cm^2^), which was even lower than the previous measurements, and lower than co 5% (*p* = 0.024), co 1% (*p* = 0.004), and the remarkably high Lam 5% condition (mean: 263.31 ± 242.52 Ω*cm^2^, *p* = 0.004). Also, Lam 1% showed a high barrier (mean: 168.91 ± 183.82 Ω*cm^2^). Lam 5% showed the highest TEER among all conditions and time points (except day 14).

#### 3.2.8. Cell Parameters (Serum Content)

**Figure 19:**
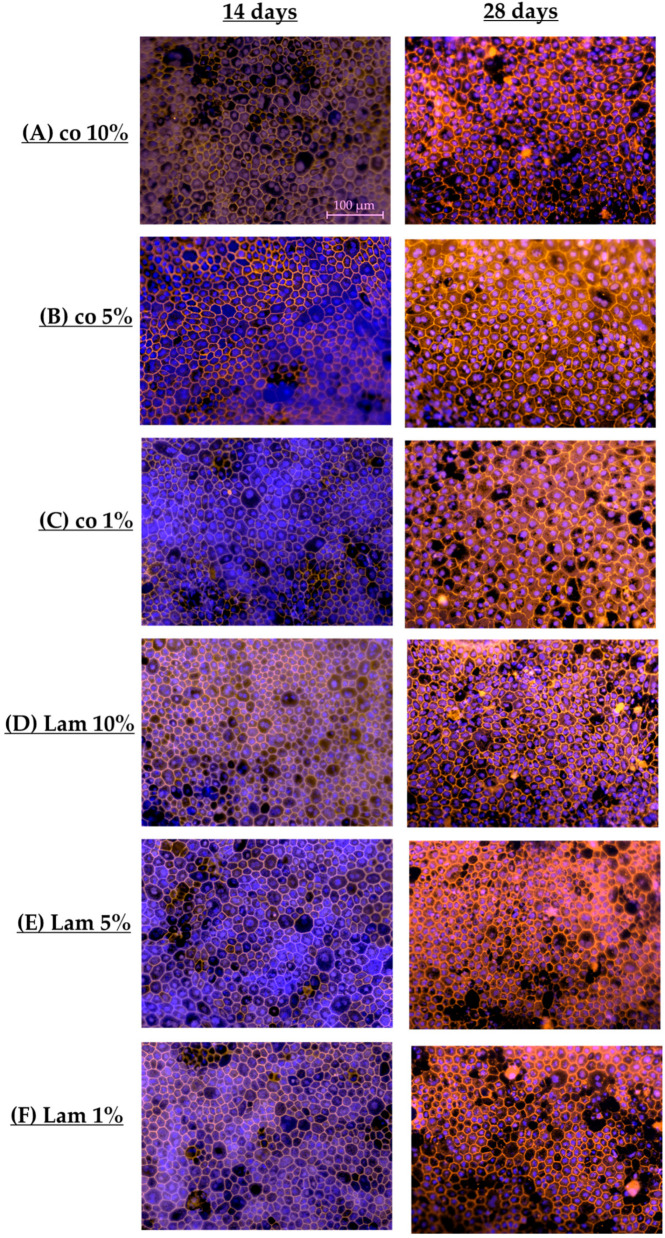
Exemplary photos. Cells used for determining morphometric parameters were stained for cell nuclei and tight junctions. Example photos for each serum condition are shown 14 and 28 days after preparation (non-coated wells (co) with 10% (**A**), 5% (**B**) and 1% (**C**) vs. laminin (Lam) with 10% (**D**), 5% (**E**) and 1% (**F**)). Objective = 20×; scale bar = 100 µm.

To examine polar single-eye RPE tight junctions and cell parameters with different serum portions, RPE cells were cultivated in Lam or non-coated (co) Transwell inserts with different serum contents (1%, 5%, 10%) for 14 or 28 days. Transwell membranes were stained for cell nuclei and tight junctions’ protein ZO-1. Images were evaluated with the CellProfiler Software (example photos shown in Figure 19) and compared with the polar RPE standard (refer Section 3.1.8). In Table 5 and Table 6, the most important parameters are listed. For all parametric data, please refer to Tables S5 and S6 in Supplementary Materials.

After 14 days of cultivation (Table 5), the mean of all polar cell cultures showed, once again, lower metric values than the polar RPE standard (e.g., area of cells is 212.67 µm^2^ compared to 267.05 µm^2^ of the polar standard) but cell number was higher (698.86 cells compared to 529.88 cells of the standard) as well as form factor (0.68 vs. 0.67 and 0.70 vs. 0.59, respectively).

According to the serum conditions, Lam 5% showed the highest cell number, whereas Lam 1% had the least cells and was closest to the polar standard. Also, Lam 1% showed the highest cell area, which was closest to the polar standard, whereas Lam 5% had the lowest one. Form factor was comparable among all conditions.

After 28 days of cultivation (Table 6), polar mean of metric data for all Transwell cultures were closer to the polar RPE standard (e.g., 483.76 cells vs. 529.88 cells of the polar standard) and the form factor was similar (0.57 vs. 0.59). Lam 10% had the lowest cell count whereas co 1% had the highest cell number closest to the polar standard, but Lam 1% and Lam 5% were close to the non-polar RPE standard. Co 1% showed the smallest cell area, which was closest to the polar standard, whereas Lam 10% had the largest while Lam 1% and Lam 5% were intermediate. Again, form factor was comparable among all conditions. Overall, all serum conditions are quite similar in the tested parameters, and a reduction in serum is feasible as RPE morphometry is not disturbed.

### 3.3. Gene Expression

Quantitative gene expression of different RPE-relevant genes was assessed with specific customized gene arrays [16]. For this, three different culture conditions were tested (co 10%, Lam 5%, Lam, 10%, each with three samples) with a cultivation time of 28 days for a full development of RPE differentiation.

All CT data were reviewed for the number of successful gene expression for each target and condition (ranging from “0” as no expression to “3” as all three samples of this condition showed gene expression). The co 10% (202), Lam 5% (192) and Lam 10% (189) were comparable in terms of successful gene expression in general.

For further evaluation, data were sorted by genes expressed in all nine samples (always expressed), genes expressed in at least two of the three replicates (mostly expressed), genes not or expressed only once in the three replicates (no expression) and genes expressed differently from zero to three times depending on the condition (varying expression), as listed in Table 7. Only genes that were expressed in all samples were included in further statistical analysis. As this was the first time single-eye RPEs were tested in polar Transwell format, different endogenous control candidates were chosen (18S rRNA, *ACTG1*, *GUSB*, *GAPDH*) while 18S rRNA was also considered as the manufacturer’s control to check if the gene array was properly produced and the assays well-conducted. As *GAPDH* showed varying expression and was not expressed in all samples, it was excluded as endogenous control and should not be used in further studies. 18S rRNA, *ACTG1*, *GUSB*, *ANXA5, APOE, BDNF, BEST1, CFH, DICER1, GUSB, HIF1A, HMOX1, IL6, MTOR, SERPING1, SOD2, TIMP1, TIMP3* and *VEGFA* were expressed in all nine samples. Targets from *ABCA4* to *VIM* (Table 7, mostly expressed) showed gene expression in most samples.

For further calculations, only the targets expressed in all nine samples were used for statistical reasons (*n* = 3). *ACTG1* was used as endogenous control as it was the most suitable (lowest CT values). Data were calculated using Thermo Fisher Connect. All ΔCT (CT of targets normalized with CT of *ACTG1* of the same sample) values are listed in Table 8. Lower ΔCT means higher gene expression. Additionally, relative quotient was calculated using co 10% as reference group (set to 1.00). The higher Rq, the more expressed the gene target compared to co 10%.

*TIMP1* was the most expressed using Lam 10%, which was also significantly different compared to co 10% (*p* = 0.037), whereas Lam 5% also showed a similar high expression but did not reach significance compared to co 10%. Of note, there were no significant findings comparing Lam 5% with Lam 10%.

Descriptively, concerning the not-significant data, Lam 10% also showed the highest gene expression for *ANXA5*, *BDNF*, *HIF1A*, *HMOX1*, *IL6*, *MTOR*, *SERPINEG1*, *TIMP3* and *VEGFA,* whereas *CFH* was least expressed.

Lam 5% did not show the highest gene expression for any gene, but the lowest gene expression was shown for *APOE*, *BDNF*, *BEST1*, *DICER1*, *HIF1A*, *HMOX1*, *IL6*, *MTOR*, *SERPINEG1*, *SOD2*, *TIMP3* and *VEGFA*.

With co 10%, gene expression was the highest for *APOE*, *BEST1*, *CFH*, *DICER1* and *SOD2,* whereas gene expression was the lowest for ANXA5. By definition, the Rq of these genes was 1.00.

## 4. Discussion

In our study, we present a polar model for adult RPE cells that is easy to perform and economical to use. More importantly, it models the human RPE rather closely and gives a reliable “working horse” for RPE research, including but not limited to AMD-related research.

We have recently published a best-practice protocol for adult porcine single-eye RPE cell culture, which has meticulously investigated several cell culture parameters for a reliable and reproducible model of adult RPEs [16]. The single-eye RPE cell culture offers a simple solution for a great drawback of conventional primary RPE cell culture—the heterogeneity of its cells. RPE cell cultures generally consist of a mixture of RPEs from different organisms, which results in a heterogeneity of the genetic background of the cells and heterogenic responses within one culture. Our protocol, however, uses the RPEs of one eye for one culture (seeded in a 12-well plate), resulting in a genetically homogeneous population within, and a genetically heterogeneous population between the cultures. While this may lead to a higher variance in biological data, it is closer to the real-life patient situation. Furthermore, the need for a higher sample size may be outweighed by a higher biological relevance of the data obtained, as effects due to clonal identity or inbred strains, as found in cell lines or mice strains, can be excluded. Our previous single-eye best-practice protocol was developed, however, for 12-well plates, hence for non-polar RPE cell culture. In this study, we have adjusted the single-eye RPE cell culture on Transwell plates for polarity and barrier function.

Our adjusted protocol clearly shows that Lam is the superior coating. In addition, the favorable serum concentration is 5%, and we also recommend 28 days for differentiation. As in our previous study, the post-mortem time is 4–5 h, and all RPE cells of one eye (ca. 150,000 cells) are seeded on one well. No further passaging is conducted to avoid de-differentiation of the cells. A major indicator of RPE barrier function is the transepithelial electric resistance. In the best conditions tested (5% serum, coating with Lam), we achieved a TEER of 263 Ω*cm^2^. This exceeds the TEER achieved by ARPE-19 cells, which usually reaches no more than 50 Ω*cm^2^ [6,9,27,28,29], although some protocols allow for about 100 Ω*cm^2^. As expected, our data do not reach the values of culture fetal RPE cells with its TEER exceeding 600–1200 Ω*cm^2^ [27,29]; however, the TEER reached by fetal tissue is not physiological for adult RPEs. Conversely, our data are in line with values achieved by adult human RPE culture (200 Ω*cm^2^) [30,31] or human-induced pluripotent stem-cell derived RPEs (200–300 Ω*cm^2^) [32,33,34], and exceeds the values reached by mixed porcine RPE cell cultures (100–200 Ω*cm^2^) [35,36] (Table 9). Of note, the value reached in our study is almost identical to TEER measured in native porcine RPE cells (262 vs. 263 Ω*cm^2^), which strongly indicates that our protocol facilitates a high degree of differentiation resembling the native tissue.

The most favorable coating is Lam, which differs from non-polar cells, were the best results were obtained with PDL [16]. In our study, Lam coating resulted in the highest TEER and the best morphological differentiation. Lam has been previously shown to be a suitable coating for RPE cells. It has been identified as enhancing RPE adhesion and differentiation when PDMS hydrogels are used, both for pluripotent stem-cell-derived and porcine RPEs, providing a basement-membrane-like environment and facilitating RPE polarization [19,38]. When used to culture pluripotent stem-cell-derived RPE cells, Lam enhances RPE maturation [38], which is in strong agreement with our findings.

Interestingly, the coating Fn exhibited the highest cell number growth and the highest ration of surviving cell culture, corresponding to previous studies which show a strong attachment of RPE cells on Fn [39], while at the same time exhibiting low differentiation with a high portion of mesenchymal cells and a low TEER, indicating that Fn supports cell proliferation and survival, but does not support RPE differentiation. Indeed, Fn has been known for a long time to be involved in RPE proliferation, migration and de-differentiation in proliferative vitreoretinopathy (PVR), actively contributing to membrane formation in the retina [40,41,42]. Our data are in accordance with these findings, and clearly discourage the use of Fn as a substrate for polarized RPE cells.

An unexpected finding was the distribution of VEGF secretion by using PDL after 28 days of cultivation, as it is a general consensus that VEGF is secreted mainly on the basolateral side of the RPE [43]. Also, not all coating conditions showed a significantly stronger basolateral secretion. Especially in serum tests, independent from serum levels, Lam coating did not show significant different apical and basolateral secretions after 7 and 28 days of cultivation. In addition, apical secretion was generally increased by Lam. A possible explanation could be the binding of VEGF on the ECM proteins, as the binding of VEGF, e.g., on Fn or Lam has been described previously in the literature [44,45]. As the coatings are applied on the basal side of the RPEs, secreted VEGF may be sequestered to a certain amount by Lam (or by PDL) coating. Furthermore, a stronger apical secretion has been shown before for several RPE cell culture models [46,47,48]. It has been claimed that VEGF is primarily secreted on the apical side until the process is saturated, after which VEGF is secreted basally [48]. As the maturation of the RPE influences the secretion of VEGF [43,49] and the ECM available may influence this as well, this may change the amount of VEGF produced as well as the saturation capacity of apical secretion, which would influence the amount of detectable VEGF in the supernatant. Conversely, it was stated that the distribution of VEGF is similar on both sides, but VEGF is removed from the apical side by the RPE more strongly than from the basolateral side. This effect, however, can only be observed after a longer period of time [50]. And indeed, many studies describing the stronger basolateral secretion of VEGF by the RPEs collect their media for 24 h or more (e.g., [43,49,51]), while we only collected for 4 h. Therefore, it is possible that the removal of VEGF on the apical side has not yet taken place and, therefore, the amount of VEGF is not yet reduced. Of note, the reversed polarity of VEGF secretion is not due to the species, as after 24 h of media collection, a stronger basolateral signal was also found for porcine RPEs [51]. Taken together, our data clearly show that Lam shows the strongest VEGF secretion (especially on the apical side). Little is known about the underlying molecular pathways on how Lam may influence VEGF secretion. However, in podocytes, Lam regulates VEGF via an increased activity of HIF-1 and protein kinase C [52]. As Lam also results in the strongest TEER, this is in alignment with previously published studies that correlates the degree of TEER with the amount of VEGF secreted [43]. Considering the potential regulation of RPE65 by Lam, to the best of our knowledge, no data are available on signal transduction pathways. However, RPE65 has been described to be regulated by ERK1/2 kinase, with a reduction in ERK1/2 also reducing RPE65 [53,54]. Lam, on the other hand, can increase or decrease ERK1/2 activation, depending on cell type and process regulated [55,56,57,58]; therefore, a regulation of RPE65 by Lam via ERK1/2 is feasible. However, more research is needed to further assess the pathways of how ECM molecules like Lam influence protein expression. In addition, Lam (in 10% serum), significantly induced TIMP-1 expression on an mRNA level. To our knowledge, this has not been shown before, as Lam so far has been described to not influence [59,60] or reduce TIMP-1 [61,62]. However, these studies were conducted in transformed cell lines, which may differ profoundly from differentiated epithelial cells.

We also established cell morphometry data for polar single-eye porcine RPE cells. Our data clearly show that the morphometry, most obviously cell number and cell size, differ profoundly between 14 days and 28 days of culture, strongly suggesting that even though an epithelial cell morphology is already seen after 14 days and the TEER between days 14 and 28 are similar, RPE cell differentiation still occurs between days 14 and 28. We therefore suggest to allow a cultivation time of 28 days for polarized RPE cells. Our data show that the morphometry of the cells corresponds to human peripheral RPEs [26].

After establishing the best coating, we compared different serum conditions. Considering all of our parameters investigated, we suggest a serum content of 5% for polar porcine single-eye RPE cell culture. While the morphological data for 1% and 5% Lam were similar after 28 days, the survival rate for the cultures after 28 days was the highest for Lam 5%. Importantly, Lam 5% displayed the highest TEER after 28 days. Furthermore, VEGF secretion was slightly higher for Lam 5% than for Lam 1%. Concerning the expression of CLDN19 and RPE65, nominal expression was slightly higher for Lam 1%; however, the high standard deviation renders these differences as biologically not very relevant and both displayed higher expression compared to Lam 10%. Taken together, a serum concentration of 5% (on Lam-coated wells after 28 days of cultivation) is recommended. Our data correspond well to previous findings, in which the addition of serum (5%) increased barrier function and tight junction protein expression [63].

Finally, we have investigated how our favorite condition (Lam 5%) differs in gene expression from the standard condition (co 10%). Our data did not exhibit any significant differences concerning gene expression between the standard condition co 10% and our favorable condition Lam 5%. However, it should be noted that there was a high variety in the expression of many of the tested genes, which is highly likely dependent on the heterogeneous genetic background of the samples. As our sample size is quite small (*n* = 3), further studies should be conducted focusing on specific genes that display a differential expression between the conditions without reaching statistical significance in this setting.

Taken together, we present a polar single-eye porcine RPE cell culture model, with best characteristics obtained with Lam as coating, 5% serum content and a 28-day cultivation time.

## 5. Conclusions

In this study, a standard operation protocol for polar single-eye retinal pigment epithelium preparation from pig eyes, for studies on barrier and polarity, was established. The optimal coating of Transwell inserts is laminin, and a reduction from serum to 5% is recommended. In addition, we recommend a culture time of 28 days to allow for high differentiation. 

## Figures and Tables

**Table 1:** Culture statistics and success rates depending on the coating. In this table, living culture rates (survived cultures/seeded cultures) and confluence culture rates (confluent cultures/survived cultures) are shown individually for the specific coating used with non-coated wells (co), Poly-ᴅ-Lysine (PDL), fibronectin (Fn), laminin (Lam) or collagen IV (CIV) and days of cultivation (7, 14, 28 days). Highest rates are marked green; lowest rates are marked light red.

**7 days**	**survived cultures/seeded cultures**	**confluent cultures/survived cultures**
co	0.86	0.47
PDL	0.91	0.32
Fn	1.00	0.46
Lam	1.00	0.69
CIV	0.96	0.39
**14 days**	**survived cultures/seeded cultures**	**confluent cultures/survived cultures**
co	0.78	0.68
PDL	0.76	0.73
Fn	1.00	0.75
Lam	0.96	0.86
CIV	0.96	0.72
**28 days**	**survived cultures/seeded cultures**	**confluent cultures/survived cultures**
co	0.71	0.78
PDL	0.76	0.83
Fn	0.93	0.85
Lam	0.88	0.96
CIV	0.88	0.82
**28 days**	**survived cultures/seeded cultures**	**confluent cultures/survived cultures**
**Total coatings**	0.81	0.83

**Table 2:** Cell parameters with different coatings at 14 days. Polar porcine single-eye retinal pigment epithelium (RPE) cells were cultured on Transwell inserts non-coated (co) or coated with laminin (Lam), Poly-ᴅ-Lysine (PDL), fibronectin (Fn) or collagen IV (CIV) for 14 days. Cell nuclei and tight junction protein claudin-19 were stained, fluorescence-imaged and evaluated with CellProfiler. Non-polar RPE standard values are shown (^1^: [16]) compared to the polar standard rates as well as the mean of the polar Transwell parameters. *n* = 9–16.

Coating	Cell Number	Area (µm^2^)	Form Factor
co	809.60	182.12	0.70
PDL	730.33	196.02	0.69
Lam	687.54	226.93	0.69
Fn	694.11	211.80	0.70
CIV	769.94	192.36	0.70
Polar RPE mean	738.30	201.85	0.70
Polar RPE standard	529.88	267.05	0.59
Non-polar RPE standard ^1^	472.31	324.25	0.68

**Table 3:** Cell parameters with different coatings at 28 days. Polar porcine single-eye retinal pigment epithelium (RPE) cells were cultured on Transwell inserts non-coated (co) or coated with laminin (Lam), Poly-ᴅ-Lysine (PDL), fibronectin (Fn) or collagen IV (CIV) for 28 days. Cell nuclei and tight junction protein claudin-19 were stained, fluorescence-imaged and evaluated with CellProfiler. Fluorescence photos were evaluated with CellProfiler. Non-polar RPE standard values are shown (^1^: [16]) compared to the polar standard rates as well as the mean of the polar Transwell parameters. *n* = 3–13.

Coating	Cell Number	Area (µm^2^)	Form Factor
Co	489.69	302.05	0.57
PDL	574.83	227.47	0.58
Lam	429.33	316.82	0.53
Fn	514.40	261.91	0.60
CIV	531.11	256.14	0.63
Polar RPE mean	507.87	272.88	0.58
Polar RPE standard	529.88	267.05	0.59
Non-polar RPE standard ^1^	472.31	324.25	0.68

**Table 4:** Culture statistics and success rates depending on the serum content. In this table, living culture rates (survived cultures/seeded cultures) and confluence culture rates (confluent cultures/survived cultures) are listed according to the specific serum content and coating used with laminin (Lam) or non-coated wells (co) with serum contents of 1, 5 or 10%, and days of cultivation (7, 14, 28 days). Highest rates are marked green; lowest rates are marked light red.

**7 days**	**survived cultures/seeded cultures**	**confluent cultures/survived cultures**
co 10%	1.00	0.72
co 5%	1.00	0.60
co 1%	1.00	0.55
Lam 10%	1.00	0.58
Lam 5%	1.00	0.55
Lam 1%	1.00	0.65
**14 days**	**survived cultures/seeded cultures**	**confluent cultures/survived cultures**
co 10%	0.78	1.00
co 5%	0.97	0.84
co 1%	1.00	0.82
Lam 10%	0.88	1.00
Lam 5%	1.00	0.81
Lam 1%	0.97	0.82
**28 days**	**survived cultures/seeded cultures**	**confluent cultures/survived cultures**
co 10%	0.75	1.00
co 5%	0.97	0.84
co 1%	0.97	0.84
Lam 10%	0.86	1.00
Lam 5%	1.00	0.81
Lam 1%	0.97	0.82
**28 days**	**survived cultures/seeded cultures**	**confluent cultures/survived cultures**
**All coatings together**	0.91	0.88

**Table 5:** Cell parameters with different serum contents at 14 days. Polar porcine single-eye retinal pigment epithelium cells were cultured on Transwell inserts coated with laminin (Lam) or non-coated Transwell inserts (co) with different serum contents (1%, 5%, 10%) for 14 days and stained for cell nuclei and claudin-19. Fluorescence photos were evaluated with CellProfiler. Non-polar RPE standard values are shown (^1^: [16]) compared to the mean of the polar Transwell parameters. *n* = 12–22.

Coating	Cell Number	Area (µm^2^)	Form Factor
Lam 1%	666.54	219.88	0.69
Lam 5%	729.37	204.83	0.71
Lam 10%	710.42	206.70	0.68
co 1%	700.85	212.39	0.70
co 5%	680.14	219.31	0.70
co 10%	705.83	212.90	0.70
Polar RPE mean	698.86	212.67	0.70
Polar RPE standard	529.88	267.05	0.59
Non-polar RPE standard ^1^	472.31	324.25	0.68

**Table 6:** Cell parameters with different serum contents at 28 days. Polar porcine single-eye retinal pigment epithelium cells were cultured on Transwell inserts coated with laminin (Lam) or non-coated Transwell inserts (co) with different serum contents (1%, 5%, 10%) for 28 days and stained for cell nuclei and claudin-19. Fluorescence photos were evaluated with CellProfiler. Non-polar RPE standard values are shown (^1^: [16]) compared to the mean of the polar Transwell parameters. *n* = 8–19.

Coating	Cell Number	Area (µm^2^)	Form Factor
Lam 1%	474.92	275.91	0.58
Lam 5%	469.67	302.04	0.58
Lam 10%	432.16	329.00	0.53
co 1%	546.17	264.99	0.61
co 5%	484.00	297.79	0.57
co 10%	495.63	295.70	0.54
Polar RPE mean	483.76	294.24	0.57
Polar RPE standard	529.88	267.05	0.59
Non-polar RPE standard ^1^	472.31	324.25	0.68

**Table 7:** Tested gene targets. Polar single-eye retinal pigment epithelium cells were cultivated using laminin (Lam) and 5% or 10% serum or non-coated Transwell inserts (co) with 10% serum. RNA was prepared after 28 days of cultivation and assessed with qPCR for listed genes. These were sorted according to the gene expression difference between all tested samples. Candidate endogenous controls are underlined. *n* = 3 for each condition; 9 samples in total.

Always Expressed	Mostly Expressed	Variable Expression	No Expression
18S rRNA *, ACTG1, ANXA5, APOE, BDNF, BEST1, CFH, DICER1, GUSB, HIF1A, HMOX1, IL6, MTOR, SERPING1, SOD2, TIMP1, TIMP3, VEGFA*	*ABCA4, ADRB2, C3, CAT, CD46, CFI, CRYAB, CST3, CTSD, CXCL8, FMO1, FN1, GAPDH, GPX4, GSS, IL6R, KDR, MAPK1, MAPK14, MMP2, MMP9, NFE2L2, NOS2, PTGS1, RDH11, RLBP1, RPE65, SERPINF1, SOD1, SPARC, TF, TLR2, TLR4, TYR, VCAM1, VIM*	*ANGPTL2, C2, C9, CASP1, CCL2, CCL5, CD59, CFB, CXCL12, FASLG, FLT1, FST, HTRA1, ICAM1, LIPC, LPL, NKAP, PTGS2, SCARB1, TGFB1, TLR3, VLDLR, VWF*	*ANGPT2, C5, CD55, COL14A1, CRP, CRYAA, CSF2, CX3CR1, CXCL10, ELN, GFAP, IGF1, IL1B, IL1R2, KIT, LEP, NOS1, PLA2G2D, TNF*

**Table 8:** Gene expression with different culture conditions. Polar single-eye retinal pigment epithelium cells were cultured using laminin (Lam) with 5% or 10% serum, or non-coated Transwell inserts (co) with 10% serum. RNA was prepared after 28 days of cultivation and assessed with qPCR for listed genes. ΔCT, mean and standard deviation (STD) were calculated. Also, relative quotient (Rq) was calculated using co 10% as the reference group (=1.00). *ACTG1* was used as endogenous control. Data were evaluated using Thermo Fisher Connect. * *p* < 0.05.

Condition	Gene	ΔCT 1	ΔCT 2	ΔCT 3	Mean	STD	Rq	*p*-Value
co 10%	*ANXA5*	5.08	6.40	6.53	6.00	0.80	1.00	1.000
	*APOE*	3.53	4.87	0.13	2.85	2.44	1.00	1.000
	*BDNF*	8.20	7.06	−0.46	4.93	4.70	1.00	1.000
	*BEST1*	7.55	6.62	5.69	6.62	0.93	1.00	1.000
	*CFH*	4.04	5.06	7.34	5.48	1.69	1.00	1.000
	*DICER1*	5.82	5.57	5.31	5.56	0.25	1.00	1.000
	*HIF1A*	7.66	7.26	5.17	6.70	1.34	1.00	1.000
	*HMOX1*	9.49	7.94	2.05	6.49	3.93	1.00	1.000
	*IL6*	7.97	13.16	6.68	9.27	3.43	1.00	1.000
	*MTOR*	7.43	7.16	4.42	6.34	1.67	1.00	1.000
	*SERPING1*	8.38	7.00	−0.58	4.93	4.82	1.00	1.000
	*SOD2*	4.61	6.13	1.34	4.02	2.45	1.00	1.000
	*TIMP1*	8.67	7.53	6.35	7.52	1.16	1.00	1.000
	*TIMP3*	4.51	2.66	3.90	3.69	0.94	1.00	1.000
	*VEGFA*	8.43	7.37	7.34	7.71	0.62	1.00	1.000
Lam 10%	*ANXA5*	5.33	3.68	3.91	4.31	0.89	2.16	0.196
	*APOE*	2.39	5.65	4.99	4.34	1.72	0.90	0.900
	*BDNF*	5.50	12.74	7.19	8.48	3.79	1.04	0.983
	*BEST1*	5.27	9.14	7.63	7.35	1.95	0.81	0.811
	*CFH*	5.95	4.96	5.31	5.41	0.50	0.66	0.301
	*DICER1*	5.84	5.93	4.80	5.52	0.63	0.97	0.927
	*HIF1A*	7.15	5.15	5.72	6.01	1.03	2.29	0.172
	*HMOX1*	5.90	12.30	7.45	8.55	3.34	1.67	0.747
	*IL6*	7.16	12.52	9.76	9.81	2.68	1.77	0.722
	*MTOR*	4.12	8.28	6.40	6.27	2.08	2.19	0.445
	*SERPING1*	5.97	9.83	7.16	7.65	1.98	1.35	0.759
	*SOD2*	5.85	6.92	6.54	6.44	0.54	0.53	0.179
	*TIMP1*	6.44	6.70	6.85	6.66	0.21	3.01	0.037 *
	*TIMP3*	4.72	2.64	2.02	3.13	1.41	1.10	0.905
	*VEGFA*	7.16	7.46	7.17	7.26	0.17	1.08	0.867
Lam 5%	*ANXA5*	5.14	5.16	3.43	4.58	0.99	1.80	0.330
	*APOE*	3.51	3.13	7.08	4.57	2.18	0.77	0.798
	*BDNF*	8.79	8.78	11.00	9.52	1.28	0.50	0.463
	*BEST1*	7.92	8.59	11.90	9.47	2.13	0.19	0.182
	*CFH*	4.42	4.90	6.68	5.34	1.19	0.70	0.556
	*DICER1*	6.92	6.12	5.11	6.05	0.91	0.67	0.398
	*HIF1A*	9.95	9.66	5.25	8.29	2.63	0.47	0.552
	*HMOX1*	8.59	7.43	12.64	9.55	2.74	0.83	0.889
	*IL6*	8.78	9.17	14.03	10.66	2.93	0.98	0.992
	*MTOR*	7.34	8.22	7.19	7.58	0.56	0.88	0.635
	*SERPING1*	7.82	9.56	11.38	9.59	1.78	0.35	0.285
	*SOD2*	5.92	6.70	7.68	6.77	0.88	0.42	0.141
	*TIMP1*	8.29	7.20	4.51	6.66	1.95	3.00	0.292
	*TIMP3*	3.39	4.55	1.94	3.29	1.31	0.98	0.975
	*VEGFA*	8.21	10.13	6.87	8.40	1.64	0.49	0.421

**Table 9:** Comparison of TEER values of different RPE model systems derived from the literature.

RPE Model System	TEER (Ohm*cm^2^)	References
ARPE-19 cells	30–100	Geisen 2006 [27]; Luo 2006 [9], Ablonczy 2011 [29], Dunn 1996 [6], Mannermaa 2010 [28]
Adult human RPE cell culture	200	Hu 1996 [30], Markert 2022 [31]
Fetal human RPE cell culture	500–1200	Geisen 2006 [27], Ablonczy 2011 [29]
hiPSC-induced RPE cell culture	200–300	Yan 2022 [32], Brandl 2014 [33], Gong 2019 [34]
Mixed porcine RPE cell culture	100–200	Terheyden 2021 [35], Toops 2014 [36]
Single-eye porcine RPE cell culture	263 (5% Serum, Lam)	This study
Natural tissue, porcine	262	Arndt 2001 [37]

## Data Availability

Data can be provided by request.

## Supplementary Materials

The following supporting information can be downloaded at: https://www.mdpi.com/article/10.3390/cells14131007/s1, Figure S1: Cell morphology on different coatings; Table S1: Culture statistics and success rates depending on the coating; Table S2: Cell parameters with different coatings at 14 days; Table S3: Cell parameters with different coatings at 28 days; Figure S2: Cell morphology depending on serum content; Table S4: Culture statistics and success rates depending on the serum content; Table S5: Cell parameters with different serum contents at 14 days; Table S6: Cell parameters with different serum content at 28 days.
