# Supplementary material for: Porcine Single-Eye Retinal Pigment Epithelium Cell Culture for Barrier and Polarity Studies
Source: Cells. 2025 Jul 1;14(13):1007. doi: 10.3390/cells14131007 (PMC12248934; doi:10.3390/cells14131007)
Supplement: Supplementary file 1 [file cells-14-01007-s001.zip › cells-3709394-supplementary.pdf]

cells

MDPI

Supplementary Materials

A - Morphology (coatings)

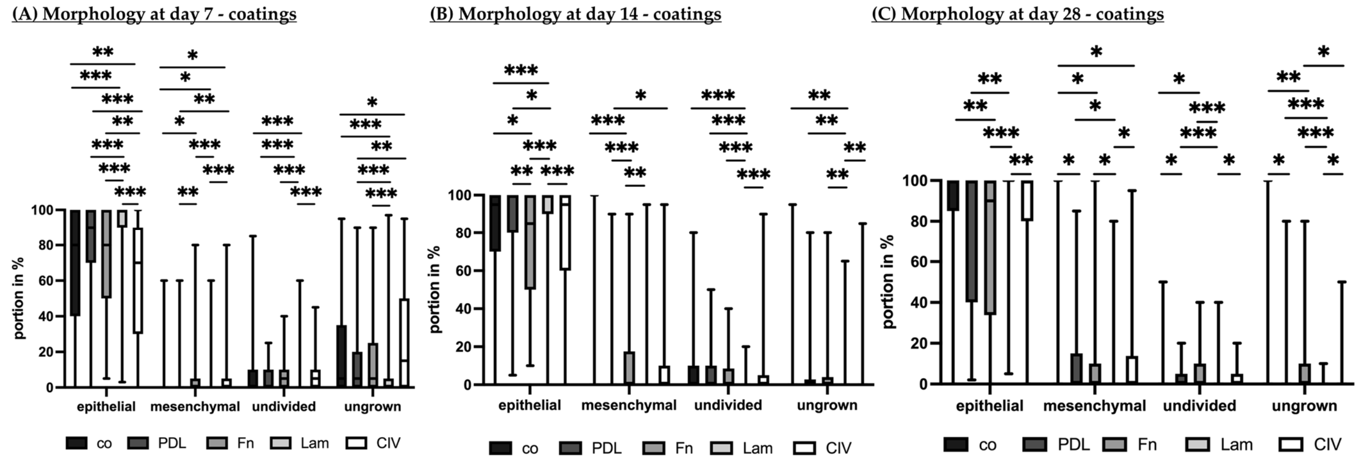

Figure S1. Cell morphology on different coatings. Porcine single-eye retinal pigment epithelium cells were cultivated on non-coated wells (co), Poly-D-Lysine (PDL), fibronectin (Fn), laminin (Lam), or collagen IV (CIV). After 7 (A), 14 (B), and 28 (C) days, the portion of epithelial cells, mesenchymal cells, undivided cells plus gaps between the cell layers of the individual wells was analyzed with bright field imaging. Data are non-parametric; median, interquartile range and range from minimum to maximum are depicted. Between each group, significances were calculated with Kruskal–Wallis test followed by Mann–Whitney test. * $p < 0.05$, ** $p < 0.01$, *** $p < 0.001$. n = 18-86.

B - Success rates (coatings)

Table S1. Culture statistics and success rates depending on the coating. In this table seeded cultures, survived cultures, dead cultures, confluent cultures, non-confluent cultures, as well as living culture rates (survived cultures/seeded cultures) and confluence culture rates (confluent cultures/survived cultures) are shown individually for the specific coating used with non-coated wells (co), Poly-D-Lysine (PDL), fibronectin (Fn), laminin (Lam) or collagen IV (CIV), and days of cultivation (7, 14, 28 days). Highest rates are marked green; lowest rates are marked light red.

**7 days**	**seeded cultures**	**survived cultures**	**dead cultures**	**confluent cultures**	**non-confluent cultures**
co	108	93	15	44	49
PDL	68	62	6	20	42
Fn	56	56	0	26	30
Lam	52	52	0	36	16
CIV	56	54	2	21	33
**14 days**	**seeded cultures**	**survived cultures**	**dead cultures**	**confluent cultures**	**non-confluent cultures**
co	108	84	24	57	27
PDL	68	52	16	38	14
Fn	56	56	0	42	14
Lam	52	50	2	43	7
CIV	56	54	2	39	15
**28 days**	**seeded cultures**	**survived cultures**	**dead cultures**	**confluent cultures**	**non-confluent cultures**
co	108	77	31	60	17
PDL	68	52	16	43	9
Fn	56	52	4	44	8
Lam	52	46	6	44	2
CIV	56	49	7	40	9

28 days	seeded cultures	survived cultures	dead cultures	confluent cultures	non-confluent cultures
All coatings together	376	305	71	252	53

7 days	survived cultures/seeded cultures	confluent cultures/survived cultures
co	0.86	0.47
PDL	0.91	0.32
Fn	1.00	0.46
Lam	1.00	0.69
CIV	0.96	0.39
**14 days**	**survived cultures/seeded cultures**	**confluent cultures/survived cultures**
co	0.78	0.68
PDL	0.76	0.73
Fn	1.00	0.75
Lam	0.96	0.86
CIV	0.96	0.72
**28 days**	**survived cultures/seeded cultures**	**confluent cultures/survived cultures**
co	0.71	0.78
PDL	0.76	0.83
Fn	0.93	0.85
Lam	0.88	0.96
CIV	0.88	0.82
**28 days**	**survived cultures/seeded cultures**	**confluent cultures/survived cultures**
All coatings together	0.81	0.83

C - Cell parameters (coatings)

Table S2. Cell parameters with different coatings at 14 days. Polar porcine single-eye retinal pigment epithelium (RPE) cells were cultured on Transwell inserts non-coated (co) or coated with laminin (Lam), Poly-ᴅ-Lysine (PDL), fibronectin (Fn), or collagen IV (CIV) for 14 days. Cell nuclei and tight junction protein claudin-19 were stained, fluorescence imaged and evaluated with CellProfiler. Non-polar RPE standard values are shown ([1]: [1]) compared to the polar standard rates as well as the mean of the polar Transwell parameters. n = 9–16.

Coating	Cell number	Area (µm^2)	Perimeter (µm)	Eccentricity	Form Factor	Radius (µm)
co	809.60	182.12	56.50	0.67	0.70	2.19
PDL	730.33	196.02	59.13	0.67	0.69	2.24
Lam	687.54	226.93	62.27	0.70	0.69	2.34
Fn	694.11	211.80	60.94	0.68	0.70	2.33
CIV	769.94	192.36	57.77	0.68	0.70	2.23
Polar RPE mean	738.30	201.85	59.32	0.68	0.70	2.26
Polar RPE standard	529.88	267.05	76.33	0.67	0.59	2.45
Non-polar RPE standard [1]	472.31	324.25	76.02	0.63	0.68	2.93

Table S3. Cell parameters with different coatings at 28 days. Polar porcine single-eye retinal pigment epithelium (RPE) cells were cultured on Transwell inserts non-coated (co) or coated with laminin (Lam), Poly-D-Lysine (PDL), fibronectin (Fn) or collagen IV (CIV) for 28 days. Cell nuclei and tight junction protein claudin-19 were stained, fluorescence imaged and evaluated with CellProfiler. Non-polar RPE standard values are shown ([1]: [1]) compared to the polar standard rates as well as the mean of the polar Transwell parameters. $n = 3–13$.

Coating	Cell number	Area (μm^2)	Perimeter (μm)	Eccentricity	Form Factor	Radius (μm)
Co	489.69	302.05	81.26	0.67	0.57	2.59
PDL	574.83	227.47	70.69	0.68	0.58	2.28
Lam	429.33	316.82	88.36	0.69	0.53	2.58
Fn	514.40	261.91	74.12	0.68	0.60	2.45
CIV	531.11	256.14	71.38	0.65	0.63	2.52
Polar RPE mean	507.87	272.88	77.16	0.68	0.58	2.48
Polar RPE standard	529.88	267.05	76.33	0.67	0.59	2.45
Non-polar RPE standard [1]	472.31	324.25	76.02	0.63	0.68	2.93

D – Morphology (serum content)

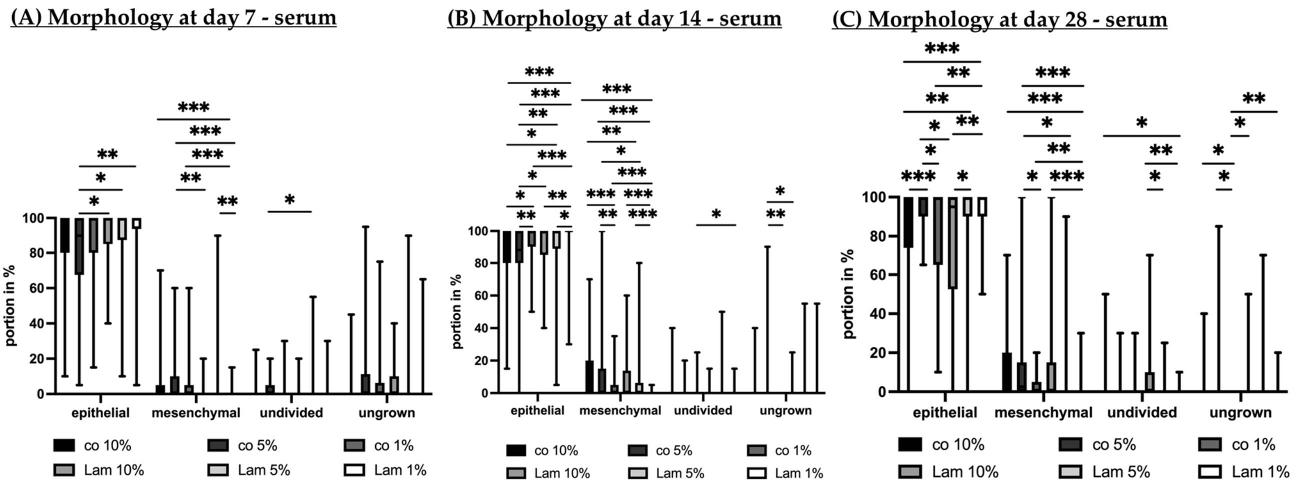

Figure S2. Cell morphology depending on serum content. Porcine single-eye retinal pigment epithelium cells were cultured on laminin (Lam), or non-coated wells (co) with different serum content (1%, 5%, 10%). After 7 (A), 14 (B) and 28 (C) days, the portion of epithelial, mesenchymal, undivided cells, and ungrown areas of the individual wells was determined with bright field microscopy photos. Data are non-parametric; median, interquartile range and range from minimum to maximum are depicted. Between each group, significances were calculated with Kruskal–Wallis test followed by Mann–Whitney test. * $p < 0.05$, ** $p < 0.01$, *** $p < 0.001$. $n = 48-54$.

E - Success rates (serum content)

Table S4. Culture statistics and success rates depending on the serum content. In this table seeded cultures, survived cultures, dead cultures, confluent cultures, non-confluent cultures as well as living culture rates (survived cultures/seeded cultures) and confluence culture rates (confluent cultures/survived cultures) are listed according to the specific serum content and coating used with laminin (Lam) or non-coated wells (co) with serum contents of 1, 5 or 10%, and days of cultivation (7, 14, 28 days). Highest rates are marked green; lowest rates are marked light red.

7 days	seeded cultures	survived cultures	dead cultures	confluent cultures	non-confluent cultures
co 10%	33	33	0	24	9
co 5%	33	33	0	20	13
co 1%	34	34	0	19	15
Lam 10%	43	43	0	25	18
Lam 5%	27	27	0	15	12
Lam 1%	35	35	0	23	12
**14 days**	**seeded cultures**	**survived cultures**	**dead cultures**	**confluent cultures**	**non-confluent cultures**
co 10%	33	26	7	26	0
co 5%	33	32	1	27	5
co 1%	34	34	0	28	5
Lam 10%	43	38	5	38	0
Lam 5%	27	27	0	22	5
Lam 1%	35	34	1	28	6
**28 days**	**seeded cultures**	**survived cultures**	**dead cultures**	**confluent cultures**	**non-confluent cultures**
co 10%	33	25	8	25	0
co 5%	33	32	1	27	5
co 1%	34	33	1	28	5
Lam 10%	43	37	6	37	0
Lam 5%	27	27	0	22	5
Lam 1%	35	34	1	28	6
**28 days**	**seeded cultures**	**survived cultures**	**dead cultures**	**confluent cultures**	**non-confluent cultures**
All coatings together	205	188	17	167	21

7 days	survived cultures/seeded cultures	confluent cultures/survived cultures
co 10%	1.00	0.72
co 5%	1.00	0.60
co 1%	1.00	0.55
Lam 10%	1.00	0.58
Lam 5%	1.00	0.55
Lam 1%	1.00	0.65
**14 days**	**survived cultures/seeded cultures**	**confluent cultures/survived cultures**
co 10%	0.78	1.00
co 5%	0.97	0.84
co 1%	1.00	0.82
Lam 10%	0.88	1.00
Lam 5%	1.00	0.81
Lam 1%	0.97	0.82
**28 days**	**survived cultures/seeded cultures**	**confluent cultures/survived cultures**
co 10%	0.75	1.00

co 5%	0.97	0.84
co 1%	0.97	0.84
Lam 10%	0.86	1.00
Lam 5%	1.00	0.81
Lam 1%	0.97	0.82
**28 days**	**survived cultures/seeded cultures**	**confluent cultures/survived cultures**
All coatings together	0.91	0.88

F - Cell parameters (serum content)

Table S5. Cell parameters with different serum contents at 14 days. Polar porcine single-eye retinal pigment epithelium cells were cultured on Transwell inserts coated with laminin (Lam), or non-coated Transwell inserts (co) with different serum contents (1%, 5%, 10%) for 14 days and stained for cell nuclei and claudin-19. Fluorescence photos were evaluated with CellProfiler. Non-polar RPE standard values are shown ([1]: [1]) compared to the mean of the polar Transwell parameters. *n* = 12–22.

Coating	Cell number	Area (µm^2)	Perimeter (µm)	Eccentricity	Form Factor	Radius (µm)
Lam 1%	666.54	219.88	62.55	0.70	0.69	2.32
Lam 5%	729.37	204.83	59.20	0.68	0.71	2.30
Lam 10%	710.42	206.70	60.84	0.68	0.68	2.26
co 1%	700.85	212.39	60.46	0.68	0.70	2.33
co 5%	680.14	219.31	61.79	0.66	0.70	2.39
co 10%	705.83	212.90	60.28	0.67	0.70	2.32
Polar RPE mean	698.86	212.67	60.85	0.68	0.70	2.32
Polar RPE standard	529.88	267.05	76.33	0.67	0.59	2.45
Non-polar RPE standard [1]	472.31	324.25	76.02	0.63	0.68	2.93

Table S6. Cell parameters with different serum content at 28 days. Polar porcine single-eye retinal pigment epithelium cells were cultured on Transwell inserts coated with laminin (Lam), or non-coated Transwell inserts (co) with different serum contents (1%, 5%, 10%) for 28 days and stained for cell nuclei and claudin-19. Fluorescence photos were evaluated with CellProfiler. Non-polar RPE standard values are shown ([1]: [1]) compared to the mean of the polar Transwell parameters. *n* = 8–19.

Coating	Cell number	Area (µm^2)	Perimeter (µm)	Eccentricity	Form Factor	Radius (µm)
Lam 1%	474.92	275.91	78.13	0.67	0.58	2.48
Lam 5%	469.67	302.04	81.11	0.67	0.58	2.61
Lam 10%	432.16	329.00	90.75	0.70	0.53	2.54
co 1%	546.17	264.99	74.03	0.66	0.61	2.48
co 5%	484.00	297.79	81.33	0.68	0.57	2.55
co 10%	495.63	295.70	86.48	0.70	0.54	2.44
Polar RPE mean	483.76	294.24	81.97	0.68	0.57	2.52
Polar RPE standard	529.88	267.05	76.33	0.67	0.59	2.45
Non-polar RPE standard[1]	472.31	324.25	76.02	0.63	0.68	2.93

Reference

1. Dörschmann, P.; Wilke, J.; Tietze, N.; Roider, J.; Klettner, A. Single-Eye Porcine Retinal Pigment Epithelium Cell Cultures—A Validated and Reproducible Protocol. *BioMed* **2025**, *5*, 7. https://doi.org/10.3390/biomed5010007.
